# Thermal diffuse scattering analysis of Ag_2_O binary system via X-ray powder diffraction

**DOI:** 10.1107/S1600576724010756

**Published:** 2025-02-01

**Authors:** Marcelo Augusto Malagutti, Binayak Mukherjee, Himanshu Nautiyal, Sebastian Bette, Narges Ataollahi, Robert Dinnebier, Paolo Scardi

**Affiliations:** a Department of Civil, Environmental, and Mechanical Engineering University of Trento Italy; b Max Planck Institute for Solid State Research Heisenbergstrasse 1 170569Stuttgart Germany; Ecole National Supérieure des Mines, Saint-Etienne, France

**Keywords:** thermal diffuse scattering, X-ray powder diffraction, density functional theory, *ab initio* molecular dynamics, silver oxide, binary systems, pair distribution functions

## Abstract

Diffuse scattering is a component of the powder pattern bearing information on the local atomic structure and disorder of crystalline materials. It is visible in the X-ray diffraction patterns of binary structures like Ag_2_O, which has a large mean squared displacement for its constituent elements. Pair distribution function (PDF) analysis is widely employed to extract this local structural information, embedded in the widths of PDF peaks. However, obtaining the PDF from experimental data requires a Fourier transform, which introduces aberrations in the transformed data due to instrument resolution, complicating the distinction between its static and dynamic components. In this work, the analysis of thermal diffuse scattering is performed directly on the X-ray powder pattern, using the traditional Rietveld method integrated with a correlated displacement model for atomic pairs. The Ag_2_O case study data were collected using synchrotron radiation at room temperature, supplemented by laboratory experiments up to 200°C. An Einstein model was used to obtain the harmonic and anharmonic force constants of the system. The force constants were also obtained via density functional theory and *ab initio* molecular dynamics simulations and showed similar values to the experiments. The analysis reveals the complex dynamic structure of Ag_2_O, characterized by high anisotropy in phonon dispersion relations and the presence of soft phonon modes, which explain the significant displacement parameters observed. The proposed approach can be easily employed for other binary or more complex systems to understand the dynamics of local forces through X-ray diffraction analysis.

## Abstract 2

This study analyses thermal diffuse scattering in X-ray powder patterns using a combined Rietveld and correlated displacement model approach applied to Ag_2_O in reciprocal space.

## 1. Introduction

The intensity of a diffraction pattern can be divided into two components: Bragg and diffuse scattering. The Bragg component arises from the coherent diffraction of X-rays with the ordered crystalline lattice, which produces well defined diffraction peaks corresponding to the periodic arrangement of atoms. The diffuse scattering instead comes from atoms deviating from perfectly periodic lattice points, due to static sources like point, line or planar defects, or crystal boundaries, as well as dynamic factors due to atomic motion within the crystal structure (Warren, 1990 ▸). Thermal diffuse scattering (TDS) specifically reflects the effects of atomic thermal vibrations on the diffractogram and provides insights into the local dynamic structure of materials.

Two methodologies are commonly used for studying the dynamics of a system via X-ray powder diffraction (XRPD): (i) total scattering analysis of the pattern and (ii) structure (Rietveld) refinement. The former option comprises the pair distribution function (PDF) analysis (Takeshi & Billinge, 2012*a* ▸) in real space and the Debye scattering equation approach (Cervellino *et al.*, 2015 ▸) employed for reciprocal space data modelling. The total scattering analysis puts diffuse and Bragg scattering on an equal footing and requires a Fourier transformation of the data (Takeshi & Billinge, 2012*a* ▸), or high computational costs to calculate the Debye scattering equation (Gelisio & Scardi, 2016 ▸) from atomic coordinates. In (ii) the Bragg and diffuse components are modelled separately, allowing the implementation in conventional routines based on Rietveld refinement (Coelho, 2018 ▸; Scardi *et al.*, 2018 ▸). The static component of the pattern can be modelled by whole powder pattern modelling (WPPM) (Scardi *et al.*, 2018 ▸), whereas the dynamic component can be modelled by analysing the collective vibration modes of the phonons via the Warren model (Warren, 1953 ▸, 1990 ▸; Beyerlein *et al.*, 2012 ▸; Perez-Demydenko, 2019 ▸) or, alternatively, via the Sakuma model (Sakuma, 1995 ▸; Scardi & Malagutti, 2024 ▸), which treats the atomic pairs as correlated oscillators. Therefore, the Sakuma TDS approach can model the effects of the short-range dynamical distortion of the system (Scardi & Malagutti, 2024 ▸), whereas the Warren TDS approach deals with longer-range dynamical features, such as phonon confinement in nanoparticles (Warren, 1953 ▸; Beyerlein *et al.*, 2012 ▸; Perez-Demydenko, 2019 ▸).

Investigating the local dynamic structure of materials provides information on the bonding mechanisms of its crystalline components, a task uniquely achievable through the Sakuma model for powder diffraction. Extending from the Rietveld unit-cell model (Coelho, 2018 ▸), which considers an average unit-cell structure for the modelling, the Sakuma approach considers its standard deviation (second moment of distribution) (Scardi & Malagutti, 2024 ▸). This allows the estimation of the correlated movement of the atoms in each coordination shell. A straightforward approach for extracting meaningful information from correlated movements is to assume an effective one-dimensional potential between any pair of atoms, as is done in extended X-ray absorption fine structure (EXAFS) analysis (Fornasini, 2001 ▸; Yang *et al.*, 1997 ▸; Fornasini *et al.*, 2001 ▸). In this case, the harmonic and anharmonic coefficients of the potential can be related to the first, second and third moments of the Fourier transform of the EXAFS data. Similar strategies have been employed for PDF analysis (Chung & Thorpe, 1997 ▸), where simple models using a harmonic potential with bond-stretching and bond-bending forces were employed.

**Figure 1:**
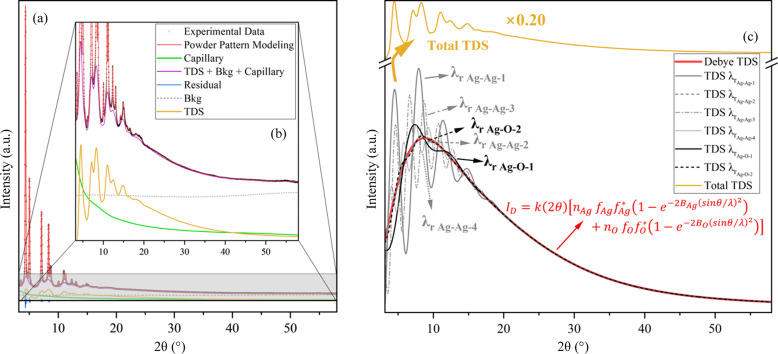
(*a*) Rietveld analysis of the XRPD pattern supported by the Sakuma TDS model. The dots represent the experimental data obtained with an SDD of 300 mm, the red line is the powder pattern modelling, the green line is the empty capillary contribution, the dashed line is the Chebyshev background, the yellow line is the Sakuma TDS contribution, the purple corresponds to all the background features (TDS + Chebyshev + empty capillary) and the blue line represents the residual of the fitting. (*b*) Zoomed-in part of the grey rectangle of (*a*). (*c*) Components of the TDS modelled by the Sakuma approach. Red corresponds to the uncorrelated Debye TDS component of equation (3). 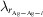 represents each of the *i*th coordination shell components of the of the TDS for the Ag–Ag pairs. 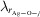 represents the TDS *j*th coordination shell for the Ag–O pairs. The yellow lines represent the total TDS contribution, which was multiplied by 0.20 in the graph for visualization purposes.

In XRPD patterns, the effect of the TDS appears as more or less broad features under the Bragg peaks (Warren, 1990 ▸), as illustrated later in Fig. 1 ▸. Its intensity scales with the number of atoms *N*, being notably lower than the *N*^2^ scaling observed for the Bragg component (Warren, 1990 ▸). Consequently, TDS is often dismissed as background noise in many XRPD studies. However, it cannot be overlooked in cases of substantial dynamic disorder such as in Ag_2_O (Wada *et al.*, 2012 ▸), whose Debye–Waller (DW) coefficients are on the order of 5 Å^2^. Consequently, Ag_2_O is the perfect case study to investigate the TDS component of binary systems.

The exotic dynamic features of Ag_2_O are attributed to the cuprite structure. The oxygen ion has tetrahedral coordination with four silver ions, forming an Ag_4/2_O tetrahedron where the silver atoms have a linear coordination with two oxygens (O—Ag—O). EXAFS analysis showed that static/dynamic deformations of these Ag_4/2_O tetrahedra are also related to the mechanism of negative thermal expansion (NTE) in Ag_2_O (Fornasini *et al.*, 2006 ▸; Beccara *et al.*, 2002 ▸; Dapiaggi *et al.*, 2003 ▸, 2008 ▸; Sanson *et al.*, 2006 ▸; Chapman & Chupas, 2009 ▸). These studies, in fact, suggest that the Ag–O nearest-neighbour distance augments slowly with temperature, whereas the Ag–Ag second-nearest-neighbour pair distance reduces (Beccara *et al.*, 2002 ▸; Sanson *et al.*, 2006 ▸). This is corroborated by other work that, using inelastic neutron scattering techniques, showed a strong anharmonic nature of phonon modes (Gupta *et al.*, 2012 ▸). In addition, *ab initio* calculations showed that transverse phonon modes are responsible for Ag_4/2_O tetrahedron bending motions (Gupta *et al.*, 2012 ▸). Other reports suggest that a static displacement of the Ag atoms is the origin of the NTE behaviour, by promoting a phase transition of the first order at around 35 K from an unknown phase to the cuprite cubic phase (Kennedy *et al.*, 2005 ▸); this phase transition was analysed via EXAFS by Sanson (2016 ▸). However, few reports employing Rietveld and PDF analysis have been published thus far to elucidate the phase transition and dynamic properties (Wada *et al.*, 2012 ▸; Dapiaggi *et al.*, 2008 ▸).

In the present article, we discuss and refine the Sakuma TDS modelling for multi-element systems and demonstrate its practical use for the Ag_2_O case. This is achieved by analysis of high-temperature XRD (HTXRD) laboratory data, room-temperature (RT) experiments from synchrotron sources, and density functional theory (DFT) and *ab initio* molecular dynamics (AIMD) simulations (Kresse & Furthmüller, 1996*b* ▸). Here we also provide tools and approaches to effectively separate the TDS from background components in XRPD patterns. Via TDS, we show that the force constants of the Ag_2_O system can be estimated using XRPD in terms of Einstein oscillators, like for EXAFS data analysis (Fornasini, 2001 ▸). This strategy is compared with a PDF analysis, which revealed that Fourier transformation and instrumental aberrations often overestimate the dynamic components of the Ag_2_O system. Through first-principles calculations, we highlight the complexity of Ag_2_O force fields, with DFT calculations unveiling a previously unknown lattice-parameter-dependent soft mode, suggesting a dynamical instability in cuprite Ag_2_O.

## 2. Materials and methods

### 2.1. X-ray powder diffraction theory

As previously mentioned, the intensity scattered from a powder sample is often written as the sum of the Bragg scattering or diffraction term *I*_B_ and the diffuse scattering term *I*_D_. Due to thermal motion, the Bragg term is reduced in intensity by the Debye factor [exp(−2*M*)] which multiplies the scattering factor *f* of the atomic species. This decrease in intensity is then transferred to the diffuse scattering component *I*_D_.

The most well known assumption employed in the work of Debye (1915 ▸) treats each atom as an independent and isotropic Einstein oscillator, having a mean square displacement MSD = 3*B*/(8π^2^) such that the Debye factor is given by
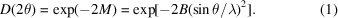
Here the wavelength of the incident radiation is represented by λ and the angle of incidence by θ, and *B* is the DW coefficient (also known as *B*_iso_ in the scalar and isotropic form). For monoatomic systems, the diffuse scattering from such uncorrelated oscillators increases by
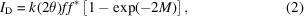
where *k*(2θ) includes terms depending on the experiments, like the Lorentz–polarization factor. 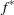 is the complex conjugate of *f*, the form factor. For the Ag_2_O case, discarding any correlations and replacing *M* from equation (1), the intensity is given by
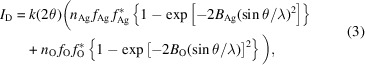
where *n*_Ag_, *n*_O_, *f*_Ag_, *f*_O_, *B*_Ag_ and *B*_O_ are the number of atoms per unit cell, atomic scattering factors and DW coefficients of Ag and O, respectively.

If the correlations in the vibration of atoms are added, considering the closest neighbours of the inner coordination shells, *I*_D_ is given by the Sakuma expression (Sakuma, 1995 ▸; Wada *et al.*, 2012 ▸):
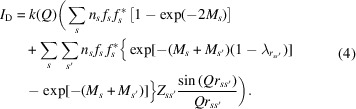
The expression above is given in reciprocal space where 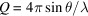, including the summation over all pairs of sites (*s* and *s*′), where *n*_*s*_ is the number of *s* atoms per unit cell and 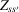 is the number of sites belonging to the *s*′ neighbour around an *s* site. In the current formulation, the equation is valid for a stoichiometric compound, but it can easily be extended to model the partial occupancy of sites (Sakuma, 1993 ▸; Scardi & Malagutti, 2024 ▸). The model originally developed by Sakuma (1995 ▸) and Wada *et al.* (2012 ▸) encompasses the correlation between atoms enclosed in one unit cell. This can be easily extended to more distant atom pairs by expanding the volume of the original unit cell and replacing *n*_*s*_ for the supercell number of atoms in the equations above (Sakuma, 1995 ▸; Scardi & Malagutti, 2024 ▸).

The correlation coefficients are defined as
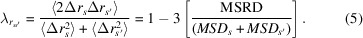
Here, 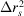 is the mean square displacement of the atom in site *s*, MSRD is the parallel component of the mean squared relative displacement for the *s*–*s*′ couple, 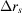 is the modulus of the displacement of an *s* atom in relation to its mean position and 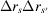 is the displacement correlation function. When displacements are uncorrelated, 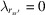, and equation (4) reduces to equation (3).

From a physical perspective, 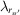 is proportional to (i) the shell coordination number (CN) and (ii) the atomic force exerted between each pair, and inversely proportional to (iii) the interatomic distance (Makhsun *et al.*, 2013 ▸; Fornasini *et al.*, 2001 ▸; Wada *et al.*, 2012 ▸). For (i), the dependence on the CN lies in the sum [equation (4)] over the number of atoms in the specific neighbour shell bonding atoms along the corresponding direction. For (ii), if the forces between the atoms are strong, the displacement correlation is correspondingly high, achieving a maximum of 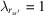 when atomic movements are totally correlated (rigid-bond model). Values from 1 to 0 correspond to acoustic modes of vibration (*i.e.* not perfectly correlated movement), whereas negative values represent optical modes. For (iii), as the atomic pairs are further apart, the forces between them decrease, eventually leading 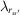 to zero. Therefore, the 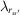 values can be employed to analyse the local dynamics of the system.

The MSRD is the second moment of the distribution of distances ρ(*r*, *T*), where ρ(*r*, *T*) corresponds to the probability of finding an atomic couple per unit of radial length, and can be also calculated from the PDF as *g*(*r*) = ρ(*r*)/ρ_0_, where ρ_0_ is the atomic number density. In EXAFS formulation (Fornasini, 2001 ▸), an effective one-dimensional pair potential is assumed in the form
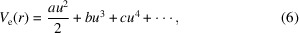
where *u* is the variation of the atomic distance concerning the potential minimum; and *a*, *b* and *c* are the force constants of the effective potential. Using a similar approach to EXAFS (Fornasini, 2001 ▸), the effective bond-stretching force constant can be estimated via the Einstein fit of the MSRD versus temperature data using the relation
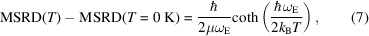
where μ is the reduced mass between the pair of atoms, *k*_B_ is the Boltzmann constant and ω_E_ is the Einstein frequency of oscillation. The effective bond-stretching force constant can be estimated as 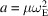. The relation between the anharmonic components and *V*_e_(*r*) is given by Fornasini (2001 ▸).

### 2.2. Experimental

The analysis of the TDS is based on XRPD data collected for 99% pure Ag_2_O powders, purchased from Sigma–Aldrich. The XRD patterns were collected using two setups. (1) A Stoe Stadi P diffractometer containing a Mythen 1K detector (DECTRIS) in Debye–Scherrer geometry, using an Mo *K*α target and a Ge111 Johan-type monochromator. The Ag_2_O powders were placed in glass capillaries of 0.15 mm radius, sealed in a vacuum and allowed to spin during the measurements. HTXRD was performed from 30 to 210°C, with temperature intervals of 20°C and a heating ramp of 5°C min^−1^. An isotherm was applied for over 30 min at each temperature step before the XRPD pattern collection. Cooling curves were also measured in steps of 50°C. (2) RT synchrotron measurements were made on the P02.1 beamline (Dippel *et al.*, 2015 ▸; Schökel *et al.*, 2021 ▸) of the Deutsches Elektronen–Synchrotron (DESY). λ = 0.20741 Å (∼60 keV) was employed and the data signal was collected by a 2D area detector [XRD 1621 from PerkinElmer (Skinner *et al.*, 2012 ▸)]. The sample was prepared in a 0.8 mm-diameter borosilicate capillary and measured using the Debye–Scherrer geometry. Two sample-to-detector distances (SDDs) were employed: 300 and 2100 mm. The 300 mm SDD measurements were used for TDS and PDF analyses due to the wider range of the wavevector modulus (*Q*). The 2100 mm SDD measurements have the highest resolution and were used for microstructural analysis.

Rietveld refinements (Rietveld, 1969 ▸) and whole powder pattern analyses were made using the *TOPAS* software (version 7; Coelho, 2018 ▸). The line profile analysis for the strain- and size-broadening effects used the WPPM (Scardi & Leoni, 2002 ▸; Scardi *et al.*, 2018 ▸) approach. A lognormal distribution of spherical domains accounted for the size broadening of the line profile. The strain broadening was accounted for using the Popa–Adler–Houska (PAH) model as a flexible approach to model anisotropic microstrain (static disorder) (Scardi *et al.*, 2018 ▸). Fundamental parameter analysis was employed to obtain the instrumental resolution function (IRF) from laboratory instrumental data by analysis of the LaB_6_ standard (NIST SRM 660c) XRD measurements. For the synchrotron measurements, a procedure developed in the literature (Dippel *et al.*, 2015 ▸; Schökel *et al.*, 2021 ▸) was used for estimating the IRF on the basis of the Thompson–Cox–Hastings pseudo-Voigt approach.

The PDF was obtained from synchrotron data, measured with an SDD of 300 mm. It used the deconvolution process available in the *TOPAS* software (Coelho, 2018 ▸). Three hat convolutions were used for smoothing the reduced structure function *F*(*Q*), and a fifth-order Chebyshev function for background. An Si 640d standard (Black *et al*., 2010 ▸) was measured to estimate the instrument’s damping function and broadening (Takeshi & Billinge, 2012*b* ▸). A maximum *Q* value *Q*_max_ = 31.8 Å^−1^ was used.

### 2.3. Computational methodology

The force constant values were compared with simulated data for the Ag_2_O system. The DFT calculations employed the *Vienna Ab initio Simulation Package* (*VASP*; Kresse & Hafner, 1994 ▸; Kresse & Furthmüller, 1996*a* ▸,*c* ▸). A 3 × 3 × 3 supercell (162 atoms) of Ag_2_O was used, accounting for the anharmonic effects. The Perdew–Burke–Ernzerhof (PBE) (Perdew *et al.*, 1996 ▸) form of the generalized gradient approximation was used to approximate the exchange-correlation energy, with scalar-relativistic PBEsol pseudopotentials (Terentjev *et al.*, 2018 ▸). A cutoff of energy 450 eV was used in the calculations, with a Gaussian charge smearing of 0.1 eV. Relaxation of the electronic degrees of freedom was performed until the change in the energy eigenvalues and the total free energy were both smaller than 10^−6^ eV. Density functional perturbation theory was used to obtain the dynamical matrix. Subsequently, the *Phonopy* (Togo & Tanaka, 2015 ▸) code was employed to perform a diagonalization of the dynamical matrix, obtaining the interatomic force constants and retrieving the phonon dispersion relations. The visualization of the simulated trajectories was done using *Ovito* (Stukowski, 2010 ▸), and *VMD* (Humphrey *et al.*, 1996 ▸) was utilized to calculate the MSD of the system.

The trajectories were generated through AIMD simulations, utilizing a 3 × 3 × 3 supercell. The calculations employed a cutoff energy of 450 eV, electronic convergence of 10^−5^ eV and Gaussian charge smearing of 0.1 eV. Sampling of the irreducible Brillouin zone was conducted at a single point using a 1 × 1 × 1 Monkhorst−Pack gamma-centred *k*-grid. AIMD calculations were carried out in a canonical (NVT) ensemble with a Nose−Hoover thermostat. Each simulation ran for 14 000 steps with a time step of 1 fs, resulting in a total simulation time of 14 ps. The simulations were performed in the temperature range 50–500 K with a step size of 50 K, including the 300 K temperature simulation.

## 3. Results and discussion

### 3.1. Synchrotron measurements

Synchrotron experiments furnish the highest quality possible for the analysis of the diffuse component. This is due to the high brilliance of synchrotron sources as well as the possibility to extend data collection to high *Q* values. For the PDF analysis, this allows a substantial reduction of truncation ripples caused by the Fourier transform (Takeshi & Billinge, 2012*a* ▸), and for TDS analysis it provides the data quality required to model the diffuse component at long *Q*, where no Bragg peaks are present.

The synchrotron diffraction Rietveld refinement using WPPM with the Sakuma TDS model is shown in Fig. 1 ▸(*a*) for a 3 × 3 × 3 supercell model [*Pn*3*m* (224), PDF database No. 41-1104]. Fig. 1 ▸(*b*) shows the amplified part with the TDS and background. The structural model did not present significant residual features, represented as a blue line in Fig. 1 ▸(*a*). *R*_wp_ approached 1% and the goodness of fit was 5. Silver carbonate (Ag_2_CO_3_, PDF database No. 04-012-6615) and silver (Ag, PDF database No. 00-004-0783) are present in the sample but correspond to less than 2% of the weight fraction (see Fig. S1 of the supporting information). To separate the background contribution from TDS modelling, an empty capillary pattern was collected prior to the measurement, being properly scaled and incorporated in the pattern modelling of the actual sample. This capillary background is represented in green in Fig. 1 ▸(*b*). In addition, a fourth-order Chebyshev polynomial was employed as a background function and proved to be constant throughout the whole 2θ range, forming the baseline of the pattern. This background is given as a grey dashed line in Fig. 1 ▸(*b*). The microstructure was modelled using the WPPM approach (Scardi *et al.*, 2018 ▸), refining the size distribution average crystallite size and standard deviation together with the PAH microstrain model. Details on the microstructure are presented in Supplementary Note 1 of the supporting information.

For 2θ angles higher than 20°, the most important contribution to the pattern originates from the TDS and the background [see Fig. 1 ▸(*b*), purple line]. This part of the diffractogram is characterized by the long tail of the uncorrelated Debye TDS component, shown in Fig. 1 ▸(*c*) as a red line, and is highly sensitive to the refined DW values. Thus, more accurate results for *B*_Ag_ and *B*_O_ were obtained by fitting the TDS in this region while constraining the DW factors to be the same as those of the Bragg part. The resulting values were *B*_Ag_ = 3.82 (4) Å^2^ and *B*_O_ = 5.8 (4) Å^2^. A comparison with the literature is given in Supplementary Note 2. With the introduction of the correlation of atomic displacements via equation (4), oscillatory features appear for 2θ < 20°, as shown in Fig. 1 ▸(*c*) – black and grey lines for Ag—O and Ag—Ag pairs, respectively. The period of these oscillations is related to the *r*_*ss*′_ values and, therefore, their amplitude tends to be higher underneath the main Bragg peaks [see purple line of Fig. 1 ▸(*b*)]. For this reason, it becomes clear that the diffuse part cannot be ignored in order to estimate the DW parameters for Ag_2_O, as it affects the intensity of the Bragg peaks.

**Figure 2:**
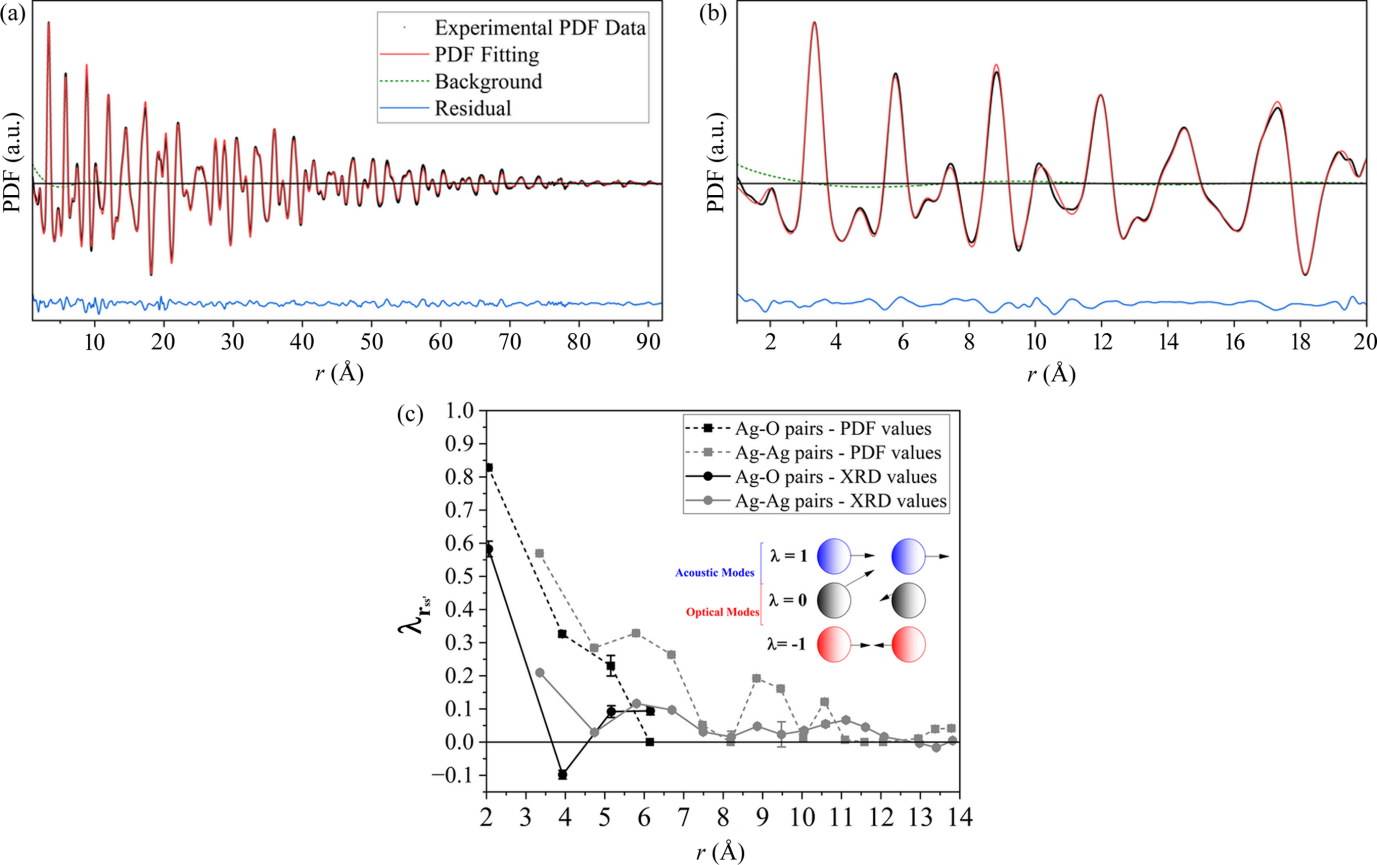
(*a*) PDF of the data collected with a 300 mm SDD, calculated by the deconvolution process available in *TOPAS* (version 7), represented as black dots. The PDF fitting is represented in red lines, the background function is in green and the blue line represents the residuals. (*b*) Zoomed-in part where the correlation modelling was applied. (*c*) Correlation coefficients as a function of the pair distance *r* obtained via the PDF analysis (dashed lines and square symbols) and the whole pattern modelling supported by the Sakuma TDS model (solid lines and circle symbols). Ag–Ag pair correlations are represented in grey and Ag–O pairs in black. The inset illustrates the correlated movement of pairs of atoms.

An alternative approach for treating diffuse components of XRPD data is via PDF analysis (Jeong *et al.*, 2003 ▸). In this methodology, the XRPD undergoes a Fourier transformation, where the local structural information is encoded in the breadths and shape of the PDF. By fitting the PDF with Gaussian bell functions, their full widths at half-maximum can be related to the MSRD and the MSD, which can be inserted into equation (5) to obtain the 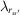 coefficients for each coordination shell (Scardi & Malagutti, 2024 ▸; Jeong *et al.*, 1999 ▸). The PDF fitting is given in Fig. 2 ▸(*a*) and the low-*r* region is highlighted in Fig. 2 ▸(*b*). The residuals show no major fitting disagreements and the overall *R*_wp_ ≃ 7.85%. The DW parameters showed values of *B*_Ag_ = 4.061 (3) Å^2^ and *B*_O_ = 9.79 (7) Å^2^, the latter being 60% higher compared with the values obtained via Sakuma-based Rietveld refinement and clearly overestimated. A cosine background function was added to correct the baseline of the data for low pair distances [*r*, represented as a green dashed line in Fig. 2 ▸(*b*)].

The 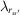 values obtained via the PDF and the Rietveld method (supported by the Sakuma TDS model) are graphically represented in Fig. 2 ▸(*c*). The 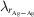 trends are similar to those for face-centred cubic (f.c.c.) metals (Jeong *et al.*, 2003 ▸) since the Ag–Ag pairs also form an f.c.c. sublattice within the Ag_2_O structure. For the Ag–O first neighbours, due to the strong ionic nature of the bonding (Gupta *et al.*, 2014 ▸), the correlation coefficient is triple that for Ag–Ag pairs, 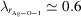, approaching a rigid-bond model.

The PDF and Sakuma TDS refinements retrieved similar trends of 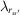 with *r*, albeit differing in magnitude. This discrepancy can be attributed to four main factors: (i) the different DW parameters obtained from each approach, (ii) the effect of an inaccurate background modelling on the Fourier transform, (iii) the 2θ resolution damping factor (Juhás *et al.*, 2018 ▸) and the IRF in the PDF data, and (iv) the termination ripples caused by the cutoff imposed by the maximum 2θ value (or *Q*_max_ value) after the transformation (Takeshi & Billinge, 2012*a* ▸). For (i), the increasing noise-to-signal ratio in the measurement at high *Q* induces a 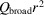 factor in the MSRD of the PDF peaks. This broadening of PDF peaks is intrinsically tied to DW values that are also obtained from the PDF peak breadths. For item (ii), the background was only accounted for by the empty capillary measurement during the Fourier transformation, disregarding other contributions. As shown in Fig. 1 ▸ (*a*), the Chebyshev contribution is of the same order of magnitude as the diffuse part, which induces the ripples observed in the green line of Fig. 2 ▸(*b*) in the PDF. In (iii), the instrumental contribution (IRF) dampens the PDF peak intensities via the equation 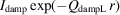

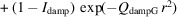
 (Beyer *et al.*, 2022 ▸), where *I*_damp_, *Q*_dampL_ and *Q*_dampG_ must be estimated using standard Si measurements (Debra & Kaiser, 2010 ▸). A further damping factor is needed for the limited *Q* resolution (Juhás *et al.*, 2018 ▸). These two effects are mainly present in the low-*r* part of the PDF, where the effects of the correlated movement are most noticeable as the sharpening of the PDF peaks. Furthermore, in (iv) the termination ripples affect the low-*r* region of the PDF as the limiting *Q*_max_ induces an imprecision in the broadening and peak position.

Modelling the local dynamic structure in reciprocal space through the Sakuma TDS approach presents the following benefits: (i) The DW factors can be estimated by both the Bragg and the TDS parts simultaneously, increasing the stability and reliability of the modelling and the resulting DW coefficient values. (ii) The instrumental components can be readily modelled by the IRF, estimated via the fundamental parameter approach using standard LaB_6_ measurements (Cheary & Coelho, 1992 ▸). In addition, the IRF contributions are only present in the line profile of the Bragg peaks and do not correlate with the diffuse part where the local dynamic information is encoded. (iii) The XRPD data can be used as they are, requiring no Fourier transformation, being free of features like the PDF peak broadening due to high noise at high *Q* (*Q*_broad_) and termination ripples at low *r* from the finite *Q*_max_ (Scardi & Malagutti, 2024 ▸).

The results presented here using synchrotron data provide the best conditions to assess the TDS and local dynamic parameters. However, synchrotron sources are not always available for routine XRPD measurements. With the following, we investigate the use of the TDS Sakuma model for laboratory data.

### 3.2. Laboratory measurements

Laboratory measurements tend to have lower beam energy and brilliance than synchrotron sources, and a larger contribution from the background is expected. For the purpose of TDS analysis, it is imperative to separate the background from the diffuse component. As already shown for the synchrotron data, the simultaneous refinement of the DW coefficients in the Bragg and TDS components helps this separation. In addition, the trends of the MSRD with temperature, obtained using the DW coefficients and 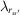, should follow the Einstein fit of equation (7). This provides an indirect way to verify the quality of fitting.

**Figure 3:**
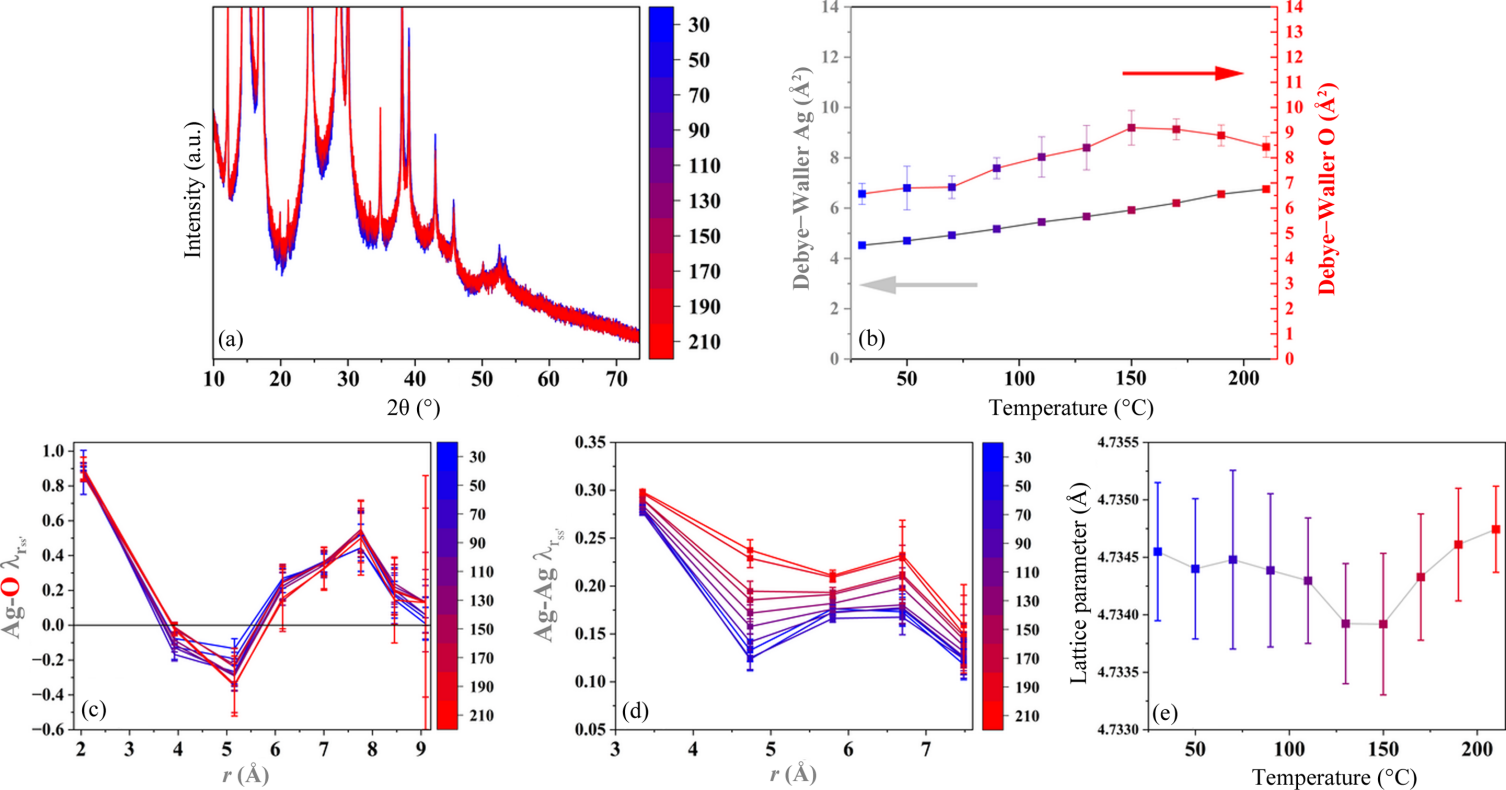
(*a*) HTXRD patterns collected with Mo *K*α radiation for the purchased Ag_2_O powders. The colours represent the temperatures (°C) in the scale on the right. (*b*) DW parameters for Ag (grey) and O (red). (*c*) Ag–O correlation coefficients versus the pair distance *r* from RT to 210°C. (*d*) Ag–Ag correlation coefficients versus the pair distance *r* from RT to 210°C. (*e*) Lattice parameter of the cuprite Ag_2_O structure for all the temperatures measured. The lines connecting the symbols are guides to the eye only.

HTXRD patterns collected using laboratory Mo *K*α radiation by heating the sample from RT to 210°C are shown in Fig. 3 ▸(*a*). Temperature increases the atomic MSD, therefore increasing the corresponding DW coefficients as indicated in Fig. 3 ▸(*b*). This also increases the TDS component intensity, foreseen by equation (4), while the Bragg peak intensities decrease due to the DW factor of equation (1). The correlation coefficients’ behaviour with temperature is shown in Fig. 3 ▸(*c*) for the Ag–O atomic pairs. The Ag–O nearest neighbours have a large correlation coefficient inside the Ag_4_O tetrahedra (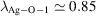), while the second and third nearest Ag–O neighbours have negative values. In addition, the displacement correlation of the Ag–O first neighbours remained constant in temperature, with no variation outside the error bars, implying that the Ag—O ionic bonds remain stiff on heating. The quality of refinement and comments on the TDS fitting are available in Supplementary Notes 3 and 4, repectively.

In contrast, the 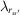 trends change significantly with temperature for the Ag–Ag pairs, as shown in Fig. 3 ▸(*d*). A likely reason for this variation is a vacancy-healing mechanism in the Ag_2_O phase. Vacancies are reported for Ag_2_O synthesized by precipitation methods (Kato, 1971 ▸), and the presence of a pure Ag phase in the pattern of Fig. S5 indicates a slightly unbalanced stoichiometry for the Ag_2_O phase. It is also known that increased temperature promotes the diffusion of Ag atoms (Kato, 1971 ▸), which can start migrating from the disordered domain boundaries of the Ag_2_O and Ag phases, filling in vacant sites. With the annealing of vacancies, the lattice dimensions tend to expand. This expansion of the lattice occurs at ∼150°C, as shown by Fig. 3 ▸(*e*), inducing a switch from NTE to positive thermal expansion (PTE) behaviour. The vacancy-healing mechanism together with a better crystallization at high temperatures also reduces the microstrain in the sample. This effect is observed in the Warren plots of Fig. S6 for some crystallographic directions. Overall, since the atoms are freer to be displaced in the direction of the vacant spaces, the correlation coefficient should be reduced. The opposite should happen when the vacancies are healed.

**Figure 4:**
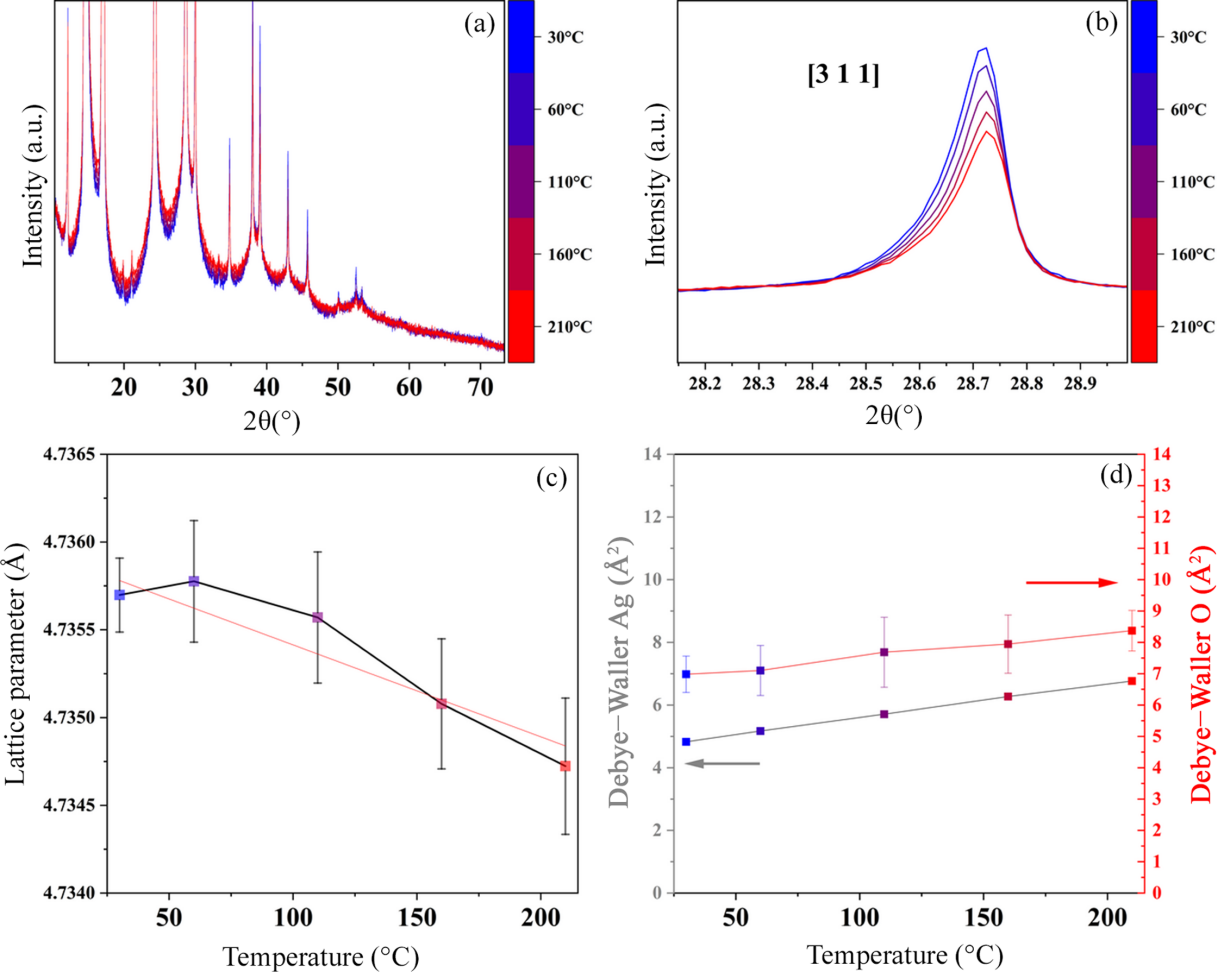
(*a*) HTXRD patterns collected with Mo *K*α radiation for the purchased Ag_2_O powders during cooling. The colours represent the temperatures (°C) in the scale on the right. (*b*) Zoom-in of the (311) plane reflection. (*c*) Refined lattice parameter. (*d*) DW parameters for Ag (grey) and O (red).

One way to eliminate the effect of microstructural evolution and vacancy healing in the analysis is to collect data in the cooling ramps. Their HTXRD patterns are shown in Fig. 4 ▸(*a*). The (311) plane reflection is given in Fig. 4 ▸(*b*), and the distinct shift towards lower angles indicates an NTE through the entire temperature range. Lattice parameters are plotted in Fig. 4 ▸(*c*) and fitted with a linear trend, giving a linear thermal expansion of 1.03 (1) × 10^−5^ K. The values for the DW parameters are similar to the heating curves, as shown in Fig. 4 ▸(*d*) and similar to what is reported in the literature (Wada *et al.*, 2012 ▸; Kennedy *et al.*, 2005 ▸; Ishikawa *et al.*, 2015 ▸).

**Figure 5:**
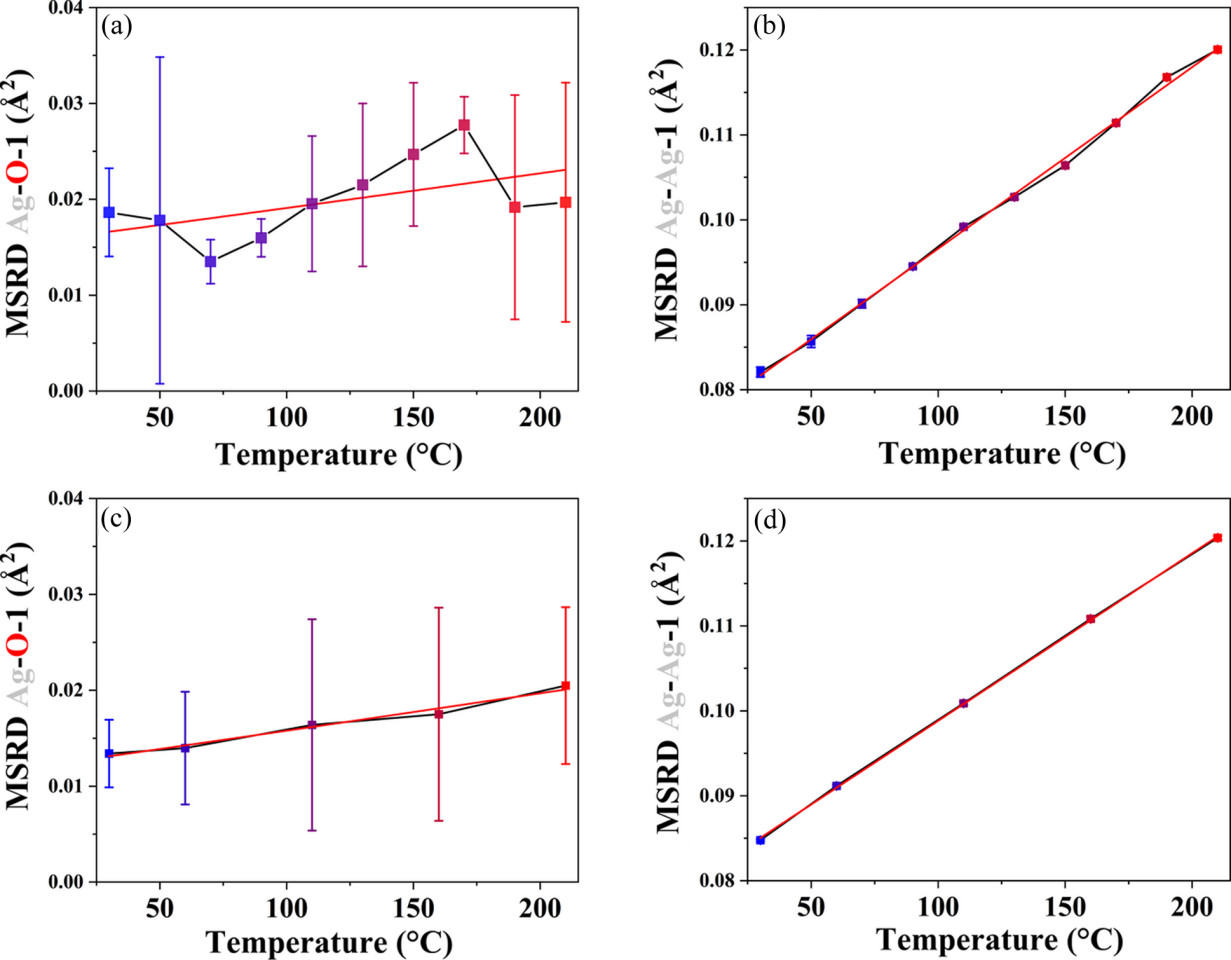
MSRD (square symbols) estimated by the HTXRD TDS fitting of (*a*) the heating ramp for the Ag–O first neighbour, (*b*) the heating ramp for the Ag–Ag first neighbour, (*c*) the cooling ramp of the Ag–O first neighbour and (*d*) the cooling ramp of the Ag–Ag first neighbour. The connecting lines are guides to the eye only. The red lines represent the Einstein model fit.

The parallel components of the MSRD, obtained by equation (5) using values of MSD from the DW coefficients and the 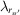 coefficients, are shown in Fig. 5 ▸ for the heating and cooling ramps. Figs. 5 ▸(*a*) and 5 ▸(*b*) show the values for Ag–O and Ag–Ag first neighbours, respectively, calculated using the heating ramp. Figs. 5 ▸(*c*) and 5 ▸(*d*) show the local dynamic parameters obtained using the cooling ramps of the Ag–O and Ag–Ag nearest neighbours. The red lines represent the Einstein model fit [equation (7)]. The 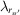 values are available in Fig. S8 for the cooling ramps in Supplementary Note 5. Here, it is possible to observe that the Ag–O–1 pair distance presents an irregular MSRD trend throughout the entire range of temperature. This is associated with the vacancy healing of the Ag_2_O lattice during heating. However, as shown in Table 1 ▸, its force constant value closely matches that of the cooling ramp in Fig. 5 ▸(*c*), indicating that the presence of vacancies has minimal impact on the TDS. Consequently, force constant values can still be accurately determined in the presence of vacancies. Likewise, the Ag–Ag–1 MSRD are not significantly different during heating or cooling. The parameters of fitting such as the Einstein temperature, calculated from the Einstein frequency (ω_E_) using the relation 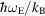, and the 0 K MSRD values are also reported in Table 1 ▸. The MSRD at *T* = 0 K approaches zero for the cooling and heating ramps for the Ag–O first-neighbour pairs, similar to that reported for EXAFS experiments (Artioli *et al.*, 2006 ▸). For the Ag–Ag pairs, this value corresponds to more than 10% of the MSRD at RT.

The force constants obtained using the Einstein model fit of the HTXRD data are higher for Ag–O and lower for Ag–Ag pairs than reported using combined XRD and EXAFS results (Artioli *et al.*, 2006 ▸), but on a similar order of magnitude. As reported by Sanson and coworkers (Artioli *et al.*, 2006 ▸), the purity of the Ag_2_O sample can induce different phase transitions at low temperatures. Therefore, different batches of Ag_2_O can give significantly different results for the force constants. This is also evident in the work of Kato (1971 ▸), where Ag_2_O was synthesized via precipitation methods. In that work, a PTE was observed instead of the NTE observed by Artioli *et al.* (2006 ▸). In addition, the Ag–Ag pairs can be divided into two types for the second nearest neighbours: the six pairs that share the same oxygen atom network (type A) and the other six that are outside of this network (Beccara *et al.*, 2002 ▸; Artioli *et al.*, 2006 ▸). We believe that diffraction alone cannot differentiate the two types of pairs, given that the PDF of the second nearest neighbour in Fig. 2 ▸ does not split into two peaks or present a significant asymmetry in order to allow the modelling of this effect by XRPD. To investigate the differences between Ag–Ag pair types in Ag_2_O and to compare the force constants obtained from HTXRD with those from first-principles simulations using DFT and AIMD, the following analysis is presented.

### 3.3. Ag_2_O simulations

**Figure 6:**
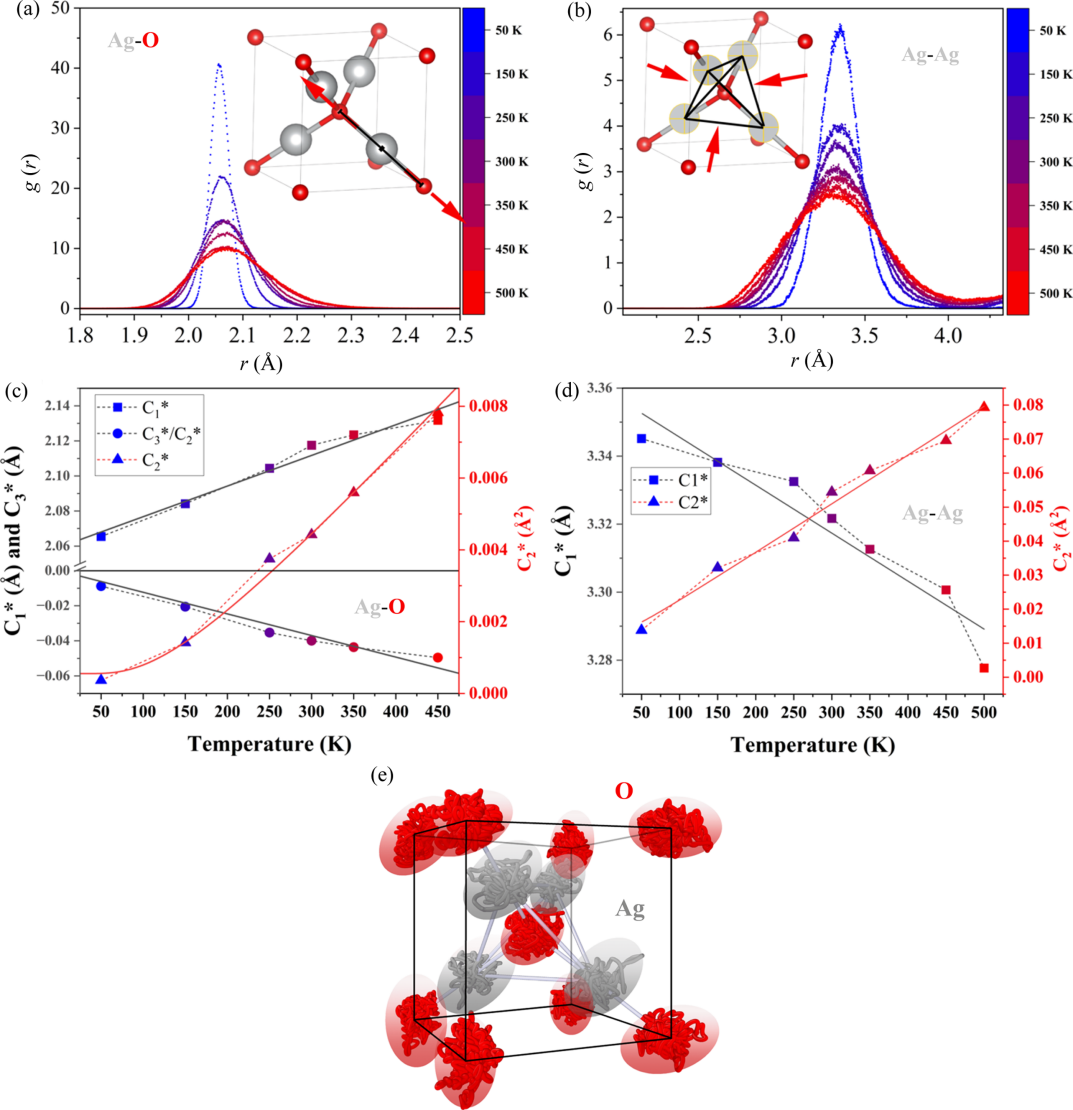
(*a*) *g*(*r*) obtained using the AIMD simulated coordinates for (*a*) Ag–O and (*b*) Ag–Ag first-neighbour pairs. The colours correspond to the scale of temperature on the right. Cumulants versus temperature for (*c*) Ag–O and (*d*) Ag–Ag first-neighbour pairs. (*e*) Trajectory of the Ag and O atoms in the unit cell simulated at 500 K. The black bars represent the unit-cell boundaries.

First-principles calculations were performed to gain insights about the local static and dynamic disorder of Ag_2_O, both at 0 K using DFT and as a function of temperature using AIMD simulations (from 50 to 500 K). From the AIMD trajectories, *g*(*r*) was calculated using the atomic coordinates at each time frame of the simulation according to procedures already established in the literature (Yang *et al.*, 1998 ▸; Fornasini *et al.*, 2001 ▸). By fitting *g*(*r*), the second cumulant 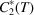 is estimated and corresponds to the parallel component of the MSRD (see Supplementary Note 6 for definitions). *g*(*r*) is represented in Figs. 6 ▸(*a*) and 6 ▸(*b*) for the Ag–O and Ag–Ag pairs, respectively, with the 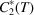 values plotted in Fig. 6 ▸(*c*) and 6 ▸(*d*), fitted using the Einstein model (red lines). The harmonic force constants obtained are 3.36 eV Å^−2^ for Ag–O and 0.6 eV Å^−2^ for Ag–Ag first neighbours (see Table 1 ▸ for fitting parameters). These values are on the same order of magnitude as reported by the HTXRD experiments in Table 1 ▸ using equation (7), and significantly higher than those reported using Wada and Ishikawa models compared in Supplementary Note 7.

Figs. 6 ▸(*a*) and 6 ▸(*b*) show the *g*(*r*) peak variation with temperature for the Ag–O and Ag–Ag nearest-neighbour pairs, respectively. While the average Ag–O separation augments with temperature, the Ag–Ag separation reduces. This is the main mechanism responsible for the NTE behaviour of Ag_2_O (Beccara *et al.*, 2002 ▸; Sanson *et al.*, 2006 ▸). The average pair distance values are given by 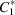, plotted in Figs. 6 ▸(*c*) and 6 ▸(*d*) for Ag–O and Ag–Ag pairs, respectively. The Ag–O pairs present a local thermal expansion^1^ of 8.5 × 10^−5^ K^−1^, while Ag–Ag experience an average thermal contraction of −4.2 × 10^−5^ K^−1^. The thermal expansion can also be calculated from the ratio 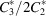 and is plotted in Fig. 6 ▸ (*c*) for Ag–O pairs. From this ratio, the anisotropic *b* parameter of equation (6) is estimated as 5.41 eV Å^−3^, in good agreement with EXAFS experiments (Artioli *et al.*, 2006 ▸). The Ag–Ag pairs present a very symmetric *g*(*r*) profile, which means that the anharmonic effects are insignificant. The *B*_Ag_ and *B*_O_ values from the AIMD simulations are 3.6 (2) and 3.6 (4) Å^2^, respectively. It is important to highlight that the effective values for Ag–Ag are placed here for direct comparison with HTXRD measurements. The correct values should consider the different intra- and internetwork Ag–Ag pairs. In Supplementary Note 6, a thorough discussion of these two types of bonding is performed and a value for the force constants is given. Additional simulations with smaller (2 × 2 × 2) and larger (4 × 4 × 4) supercells are given in the same supplementary note, highlighting that a 3 × 3 × 3 simulation box is a good compromise between precision and speed for simulating the system.

Here we observe that the proximity of values using the Einstein model for the HTXRD experimental and AIMD-simulated data confirms that the TDS model is a valid tool for investigating the local dynamics of Ag_2_O. However, we believe that the absolute value of the force constants cannot be attained with enough precision with the current setup. Two reasons can be given: one is that the one-dimensional effective potential of equation (6) tends to be a very simple modelling that might not capture all the complex features of the Ag_2_O system, and the second is that the XRD might not have sufficient resolution to capture the subtle details from the diffuse components. A visual representation of the complexity of the Ag_2_O system is represented by the vibration of the ions shown in Fig. 6 ▸(*e*), corresponding to the trajectory of Ag and O atoms over the entire simulation time at 500 K. In particular, the Ag atoms are found to have displacement patterns in the shape of ellipsoids with the long axis perpendicular to the O—Ag—O bonds, in agreement with previous experimental measurements (Kennedy *et al.*, 2005 ▸). This suggests that non-central forces might be present for the Ag_2_O system and cannot be modelled by equation (6).

Further complexity in the static component of the Ag_2_O system can be understood via DFT. A quantitative measure of the anisotropy, the log-Euclidean anisotropy parameter (*A*^L^) (Kube, 2016 ▸), can be obtained from the compliance tensor, calculated by DFT. For Ag_2_O, *A*^L^ = 3.2, a value considered to be quite high for the symmetric cubic structure. For comparison, the highest calculated *A*^L^ reported by Kube & Jong (2016 ▸) for a cubic structure is 5.3 for SrVO_3_. This anisotropy is also reflected in the calculated interatomic force constant tensor, whose values for nearest neighbours vary according to direction (see Supplementary Note 8) and contain non-zero off-diagonal terms. As expected, the IFCs for the first Ag–O pairs are found to be significantly stronger than those for the first Ag–Ag pair, in agreement with the correlations obtained from the TDS modelling of the XRD data, where the value for 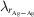
is half that of 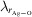. However, note that this large anisotropy is somewhat at odds with the use of a simple one-dimensional potential such as equation (6) – in particular, the presence of off-diagonal terms in the IFC tensors suggests that a simple central-force model for the interactions in Ag_2_O has certain limitations.

**Figure 7:**
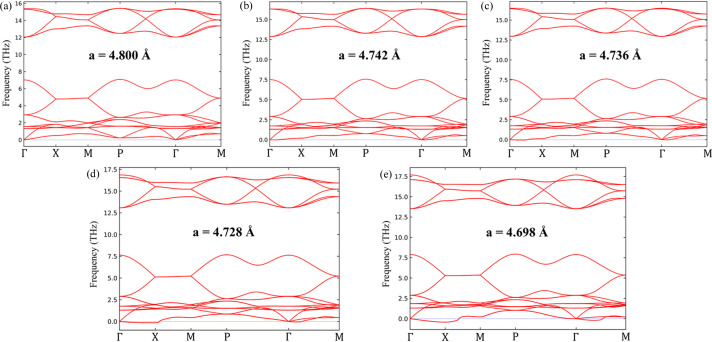
Phonon dispersions for a range of lattice parameters: (*a*) 4.800 Å, (*b*) 4.742 Å, (*c*) 4.736 Å, (*d*) 4.728 Å and (*e*) 4.698 Å.

Further details about the nature of the static disorder in Ag_2_O are revealed from the phonon dispersion obtained from the IFCs. The phonon dispersions (Fig. 7 ▸) are computed by constraining the lattice parameter of Ag_2_O to a range of values between ∼4.8 and ∼4.7 Å (the ground state values of the lattice parameter obtained using a simple PBE functional and a modified PBEsol functional, respectively) and show that the lowest-energy acoustic mode becomes soft (imaginary frequencies, represented as negative) along the Γ-X and Γ-M directions, as the lattice parameter drops below 4.736 Å. Additionally, the calculated mode Grueneisen parameter (see Supplementary Note 8, Fig S15) shows high anharmonicity at frequencies <2 THz. These soft modes, which have not been observed in previous calculations of the phonon dispersion of Ag_2_O using a simple PBE functional (Li, 2022 ▸), correspond to non-restorative forces on the atoms which make the structure dynamically unstable. The experimental lattice parameters as shown in Fig. 4 ▸(*d*) lie between 4.7345 and 4.7360 Å, which indicates that the system is quite close to dynamical instability. As such, the associated structural softness (IFCs approaching 0) can be responsible for the large displacement vibrations of the ions, which give rise to the large TDS. Additionally, the presence of the soft modes raises the question of a potential phase transition in Ag_2_O, as already reported in the literature (Sanson, 2016 ▸; Kennedy *et al.*, 2005 ▸). Hence, here we presented the main barriers to understanding the Ag_2_O local dynamic behaviour through TDS approaches. We believe that further advancements in the theory must be performed for the full comprehension of these complex systems, especially involving non-central forces.

## 4. Conclusions

The TDS modelling of binary phases using XRPD is demonstrated in this work using the case of Ag_2_O measured in synchrotron and laboratory facilities. The proposed approach based on Rietveld refinement integrated with the Sakuma TDS model provides access to correlation displacement coefficients for atomic pairs in the inner coordination shells. The Einstein model was used for estimating force constants assuming a one-dimensional effective potential, providing harmonic force constants on the order of 2 and 0.4 eV Å^−2^ for Ag–O and Ag–Ag first neighbours, respectively. Compared with first-principles studies (DFT and AIMD), the force constants are of the same order of magnitude. First-principles calculations point to a complex interplay between static and dynamic components of disorder as the origin of the large TDS. This includes (i) the vibrations of ions with large displacements about their lattice sites, (ii) the anisotropic interactions between Ag–Ag and Ag–O pairs (also responsible for the NTE) leading to distortions of the Ag_4/2_O tetrahedra, and (iii) distortions associated with the proximity of the Ag_2_O structure to dynamical instability. The present work thus explains the atypical vibrational behaviour of Ag_2_O while establishing a novel framework to analyse similar systems with large TDS using XRPD.

## 5. Related literature

The following references are only cited in the supporting information: Dalba *et al.* (1998 ▸); Ishikawa *et al.* (2015 ▸); Makhsun *et al.* (2014 ▸); Takeshi & Billinge (2012*c* ▸); Wada *et al.* (2012 ▸); Yoshiasa *et al.* (1997 ▸).

## Supplementary Material

Word document (Supplementary information containing auxiliary diffraction paterns, microscopy results, theory, etc.). DOI: 10.1107/S1600576724010756/nb5390sup1.pdf

## Figures and Tables

**Table 1:** Estimated force constants for the Ag–O and Ag–Ag first nearest neighbours using equation (7) for the heating ramp, cooling ramp, AIMD simulations and DFT simulations θ_E_ is the Einstein temperature calculated by 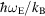.

Parameter	Ag–O (heating ramp)	Ag–O (cooling ramp)	Ag–O (AIMD)	Ag–O (DFT)	Ag–Ag (heating ramp)	Ag–Ag (cooling ramp)	Ag–Ag (AIMD)	Ag–Ag (DFT)
ω_E_ (THz)	39.7	38.3	48.3		8.48	8.83	10.3	
MSRD at *T* = 0 K (Å^2^)	0.004 (1)	0	0.0043		0.0164 (2)	0.0248 (1)	0.008	
θ_E_ (K)	303	292	370		65	67	80	
Force constant *a* using the Einstein model (eV Å^−2^)	This work: 2.27	2.12	3.37	3.9 (0 K)	This work: 0.40	0.44	0.6	0.3 (0 K)
	Artioli *et al.* (2006 ▸): 5.93†				Artioli *et al.* (2006 ▸): 0.164			
	Beccara *et al.* (2002 ▸): 5.77							

† Estimated from the Einstein frequencies available in the references.

## Data Availability

Data are available upon request.

## Footnotes

1 The local thermal expansion is estimated by the slope of the linear fits of Figs. 6 ▸(*c*) and 6 ▸(*d*) divided by  (*T* = 0 K), called *R*_0_.

## References

[bb1] Artioli, G., Dapiaggi, M., Fornasini, P., Sanson, A., Rocca, F. & Merli, M. (2006). *J. Phys. Chem. Solids*, **67**, 1918–1922.

[bb2] Beccara, S., Dalba, G., Fornasini, P., Grisenti, R., Sanson, A. & Rocca, F. (2002). *Phys. Rev. Lett.* **89**, 025503. 10.1103/PhysRevLett.89.025503 12097002

[bb3] Beyer, J., Roth, N. & Brummerstedt Iversen, B. (2022). *Acta Cryst.* A**78**, 10–20. 10.1107/S2053273321011840 34967326

[bb4] Beyerlein, K. R., Leoni, M. & Scardi, P. (2012). *Acta Cryst.* A**68**, 382–392. 10.1107/S0108767312009853 22514070

[bb12] Black, D. R., Windover, D., Henins, A., Gil, D., Filliben, J. & Cline, J. P. (2010). *Powder Diffr.* **25**, 187–190.

[bb5] Cervellino, A., Frison, R., Bertolotti, F. & Guagliardi, A. (2015). *J. Appl. Cryst.* **48**, 2026–2032.

[bb6] Chapman, K. W. & Chupas, P. J. (2009). *Chem. Mater.* **21**, 425–431.

[bb7] Cheary, R. W. & Coelho, A. (1992). *J. Appl. Cryst.* **25**, 109–121.

[bb8] Chung, J. S. & Thorpe, M. F. (1997). *Phys. Rev. B*, **55**, 1545–1553.

[bb9] Coelho, A. A. (2018). *J. Appl. Cryst.* **51**, 210–218.

[bb101] Dalba, G., Fornasini, P., Grisenti, R., Pasqualini, D., Diop, D. & Monti, F. (1998). *Phys. Rev. B*, **58**, 4793–4802.

[bb10] Dapiaggi, M., Kim, H. J., Božin, E. S., Billinge, S. J. L. & Artioli, G. (2008). *J. Phys. Chem. Solids*, **69**, 2182–2186.

[bb11] Dapiaggi, M., Tiano, W., Artioli, G., Sanson, A. & Fornasini, P. (2003). *Nucl. Instrum. Methods Phys. Res. B*, **200**, 231–236.

[bb13] Debye, P. (1915). *Annal. Phys.* **351**, 809–823.

[bb14] Dippel, A.-C., Liermann, H.-P., Delitz, J. T., Walter, P., Schulte-Schrepping, H., Seeck, O. H. & Franz, H. (2015). *J. Synchrotron Rad.* **22**, 675–687. 10.1107/S1600577515002222 PMC4416682 25931084

[bb15] Fornasini, P. (2001). *J. Phys. Condens. Matter*, **13**, 7859–7872.

[bb16] Fornasini, P., Dalba, G., Grisenti, R., Purans, J., Vaccari, M., Rocca, F. & Sanson, A. (2006). *Nucl. Instrum. Methods Phys. Res. B*, **246**, 180–183.

[bb17] Fornasini, P., Monti, F. & Sanson, A. (2001). *J. Synchrotron Rad.* **8**, 1214–1220. 10.1107/s0909049501014923 11679775

[bb18] Gelisio, L. & Scardi, P. (2016). *Acta Cryst.* A**72**, 608–620. 10.1107/S2053273316014881 27809200

[bb19] Gupta, M. K., Mittal, R., Chaplot, S. L. & Rols, S. (2014). *J. Appl. Phys.* **115**, 133507.

[bb20] Gupta, M. K., Mittal, R., Rols, S. & Chaplot, S. L. (2012). *Physica B*, **407**, 2146–2149.

[bb21] Humphrey, W., Dalke, A. & Schulten, K. (1996). *J. Mol. Graph.* **14**, 33–38. 10.1016/0263-7855(96)00018-5 8744570

[bb22] Ishikawa, Y., Danilkin, S. A., Avdeev, M., Kamiyama, T. & Sakuma, T. (2015). *JPS Conf. Proc.* **8**, 031016.

[bb102] Ishikawa, Y., Sakuma, T., Hashimoto, T., Xianglian, Kamishima, O. & Danilkin, S. A. (2015). *Atom Indones.* **41**, 1–5.

[bb23] Jeong, I. K., Heffner, R. H., Graf, M. J. & Billinge, S. J. L. (2003). *Phys. Rev. B*, **67**, 104301.

[bb24] Jeong, I. K., Proffen, T., Mohiuddin-Jacobs, F. & Billinge, S. J. L. (1999). *J. Phys. Chem. A*, **103**, 921–924.

[bb25] Juhás, P., Louwen, J. N., van Eijck, L., Vogt, E. T. C. & Billinge, S. J. L. (2018). *J. Appl. Cryst.* **51**, 1492–1497.

[bb26] Kato, Y. A. (1971). *J. Am. Ceram. Soc.* **55**, 2–5.

[bb27] Kennedy, B. J., Kubota, Y. & Kato, K. (2005). *Solid State Commun.* **136**, 177–180.

[bb28] Kresse, G. & Furthmüller, J. (1996*a*). *Comput. Mater. Sci.* **6**, 15–50.

[bb30] Kresse, G. & Furthmüller, J. (1996*c*). *Phys. Rev. B*, **54**, 169–186. 10.1103/physrevb.54.11169 9984901

[bb29] Kresse, G. & Furthmüller, J. (1996*b*). *Phys. Rev. B*, **54**, 11169–11186. 10.1103/physrevb.54.11169 9984901

[bb31] Kresse, G. & Hafner, J. (1994). *Phys. Rev. B*, **49**, 14251–14269. 10.1103/physrevb.49.14251 10010505

[bb32] Kube, C. M. (2016). *AIP Adv.* **6**, 095209.

[bb33] Kube, C. M. & de Jong, M. (2016). *J. Appl. Phys.* **120**, 165105.

[bb70] Li, Y. (2022). *Front. Phys.* **10**, 1107783.

[bb103] Makhsun, T. Hashimoto, T. Sakuma, H. Takahashi, O. Kamishima, N. Igawa, S.A. Danilkin, (2014). *J. Phys. Soc. Jpn*, **83**, 6–9.

[bb34] Makhsun, T., Sakuma, T., Kartini, E., Sakai, R., Takahashi, H., Igawa, N. & Danilkin, S. A. (2013). *Atom Indones.* **39**, 8–12.

[bb35] Perdew, J. P., Burke, K. & Ernzerhof, M. (1996). *Phys. Rev. Lett.* **77**, 3865–3868. 10.1103/PhysRevLett.77.3865 10062328

[bb36] Perez-Demydenko, C. (2019). PhD thesis, University of Trento, Italy.

[bb37] Rietveld, H. M. (1969). *J. Appl. Cryst.* **2**, 65–71.

[bb39] Sakuma, T. (1993). *J. Phys. Soc. Jpn*, **62**, 4150–4151.

[bb40] Sakuma, T. (1995). *Bull. Electrochem.* **11**, 57.

[bb41] Sanson, A. (2016). *J. Phys. Chem. Solids*, **95**, 114–118.

[bb42] Sanson, A., Rocca, F., Dalba, G., Fornasini, P., Grisenti, R., Dapiaggi, M. & Artioli, G. (2006). *Phys. Rev. B*, **73**, 214305.

[bb43] Scardi, P., Azanza Ricardo, C. L., Perez-Demydenko, C. & Coelho, A. A. (2018). *J. Appl. Cryst.* **51**, 1752–1765.

[bb44] Scardi, P. & Leoni, M. (2002). *Acta Cryst.* A**58**, 190–200. 10.1107/s0108767301021298 11832590

[bb45] Scardi, P. & Malagutti, M. A. (2024). *Cryst. Growth Des.* **24**, 4380–4392.

[bb47] Schökel, A., Etter, M., Berghäuser, A., Horst, A., Lindackers, D., Whittle, T. A., Schmid, S., Acosta, M., Knapp, M., Ehrenberg, H. & Hinterstein, M. (2021). *J. Synchrotron Rad.* **28**, 146–157. 10.1107/S1600577520013223 PMC7842216 33399563

[bb48] Skinner, L. B., Benmore, C. J. & Parise, J. B. (2012). *Nucl. Instrum. Methods Phys. Res. A*, **662**, 61–70.

[bb49] Stukowski, A. (2010). *Modell. Simul. Mater. Sci. Eng.* **18**, 015012.

[bb50] Takeshi, E. & Billinge, S. J. L. (2012*a*). Editors. *Underneath the Bragg peaks*, Pergamon Materials Series, Vol. 16, pp. 259–295. Pergamon.

[bb51] Takeshi, E. & Billinge, S. J. L. (2012*b*). Editors. *Underneath the Bragg peaks*, Pergamon Materials Series, Vol. 16, pp. 55–111. Pergamon.

[bb104] Takeshi, E. & Billinge, S. J. L. (2012*c*). Editors. *Underneath the Bragg peaks*, Pergamon Materials Series, Vol. 16, pp. 325–369. Pergamon.

[bb52] Terentjev, A. V., Constantin, L. A. & Pitarke, J. M. (2018). *Phys. Rev. B*, **98**, 214108.

[bb53] Togo, A. & Tanaka, I. (2015). *Scr. Mater.* **108**, 1–5.

[bb105] Wada, T., Sakai, R., Itakura, M., Hayashi, Y., Watanube, M., Makhsun, T. Sakuma, T. Takahashi, H. & Danilkin, S. A. (2012). *Solid state ionics*, pp. 563–568. World Scientific.

[bb54] Wada, T., Sakuma, T., Sakai, R., Uehara, H., Xianglian, Takahashi, H., Kamishima, O., Igawa, N. & Danilkin, S. A. (2012). *Solid State Ionics*, **225**, 18–21.

[bb56] Warren, B. E. (1953). *Acta Cryst.* **6**, 803.

[bb57] Warren, B. E. (1990). *X-ray diffraction*, p. 398. Courier Corporation.

[bb58] Yang, D. S., Fazzini, D. R., Morrison, T. I., Tröger, L. & Bunker, G. (1997). *J. Non-Cryst. Solids*, **210**, 275–286.

[bb59] Yang, D. S., Joo, S. K. & Hilbrandt, N. (1998). *J. Korean Phys. Soc.* **33**, 59–65.

[bb106] Yoshiasa, A., Koto, K., Maeda, H. & Ishii, T. (1997). *Jpn. J. Appl. Phys.* **36**, 781.

