# Supplementary material for: Thermal diffuse scattering analysis of Ag2O binary system via X-ray powder diffraction
Source: J Appl Crystallogr. 2025 Feb 1;58(Pt 1):18–30. doi: 10.1107/S1600576724010756 (PMC11798509; doi:10.1107/S1600576724010756)
Supplement: Supplementary file 1 [file j-58-00018-sup1.pdf]

JOURNAL OF
APPLIED
CRYSTALLOGRAPHY

Volume 58 (2025)

Supporting information for article:

Thermal diffuse scattering analysis of Ag_2O binary system via X-ray powder diffraction

Marcelo Augusto Malagutti, Binayak Mukherjee, Himanshu Nautiyal, Sebastian Bette, Narges Ataollahi, Robert Dinnebier and Paolo Scardi

Supplementary Note 1: High-resolution data

The microstructural features and estimation of impurities in the Ag_2O sample are better accessed by high-resolution synchrotron measurements using the data collected with SDD of 2100 mm. The whole pattern modelling, aided by the Sakuma TDS approach, is given in Figure S 1(a). This pattern presents high asymmetry in the Bragg peaks [Figure S 1(b)], which indicates the presence of a double-sized distribution of Ag_2O phases with different lattice parameters. One of the phases, called Ag_2O-1, has its line profile profiles modelled by the Popler-Adler-Houska (PAH) microstrain model [1], with no crystallite size broadening contribution. This phase has a lattice parameter of 0.47401(2) nm. Warren plots for several plane directions are shown in Figure S 1(c). The other phase has an average crystallite size of 90(11) nm and lattice parameters of 0.47236(5) nm. The presence of the silver carbonate and Ag phases are indicated in the peaks of Figure S 1(d).

TEM and DLS measurements were acquired to infer the presence of a double-size distribution, and are shown in Figure S 2, Figure S 3, and Figure S 4. DLS analysed solely the agglomerated size of the Ag_2O nanoparticles, while TEM revealed the presence of small Ag_2O nanocrystals for the bimodal distribution. Nevertheless, the Sakuma TDS model assumed average lattice parameters for its refinement and dummy microstructural models were employed in the SDD 300 mm data analysis due to the lower resolution.

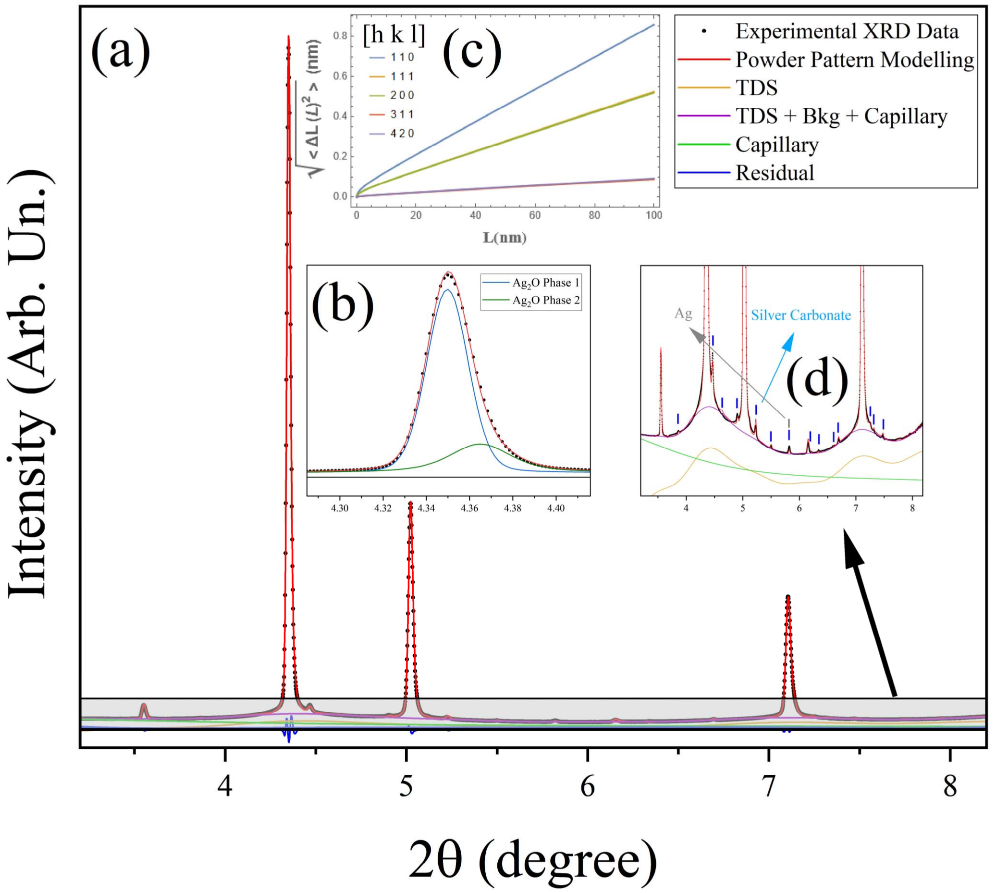

Figure S 1. (a) XRPD pattern modelling of the data collected at an SDD of 2100 mm. The dots represent the experimental data, the red line is the powder pattern modelling, the yellow line is the TDS model, in green represents the empty capillary pattern, and the purple is the combined background contribution from the Chebyshev polynomials, TDS, and empty capillary measurements. The blue line is the residual of the fitting. (b) Profile contribution for both phases of Ag_2O 1 (big crystallite size) and Ag_2O 2 (small crystallite size). (c) The Warren plot for the Ag_2O 1 phase. (d) The zoomed part is represented by the grey rectangle in item a.

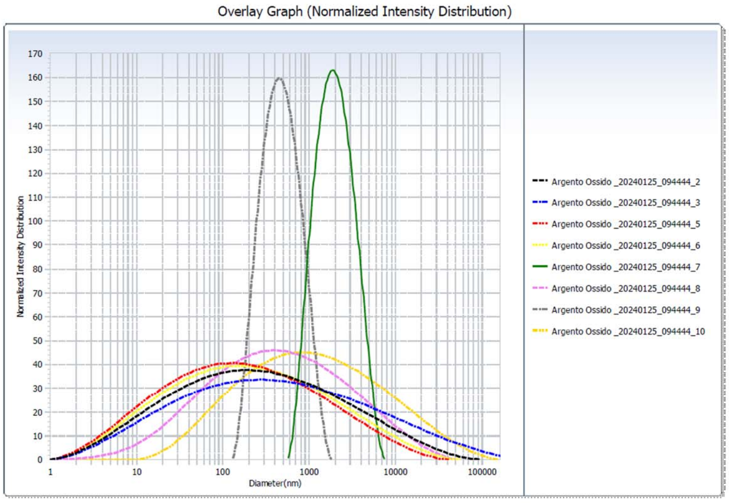

No	Data	Repet. No	pH	Ave.Diameter(nm)	PD	Mean.(nm)	D (10%) (nm)	D (50%) (nm)	D (90%) (nm)
1	Argento Ossido _20240125_094444_2	2	NA	918.9	8.700	1962.3	12.7	218.8	4514.2
2	Argento Ossido _20240125_094444_3	3	NA	2086.4	22.653	5159.5	14.9	340.4	10525.7
3	Argento Ossido _20240125_094444_5	5	NA	529.9	4.629	990.7	10.4	144.3	2387.9
4	Argento Ossido _20240125_094444_6	6	NA	653.7	5.678	1283.6	11.4	172.2	3056.8
5	Argento Ossido _20240125_094444_7	7	NA	3259.2	-65.952	2215.1	992.8	1910.7	3803.9
6	Argento Ossido _20240125_094444_8	8	NA	732.5	3.967	1831.1	35.8	401.6	4627.3
7	Argento Ossido _20240125_094444_9	9	NA	765.8	-1.982	528.3	232.0	454.5	913.6
8	Argento Ossido _20240125_094444_10	10	NA	1632.5	7.249	4893.5	90.3	947.9	12090.0
Average :				1322.4	-1.882	2358.0	175.0	573.8	5239.9

Figure S 2. SLS results for Ag_2O.

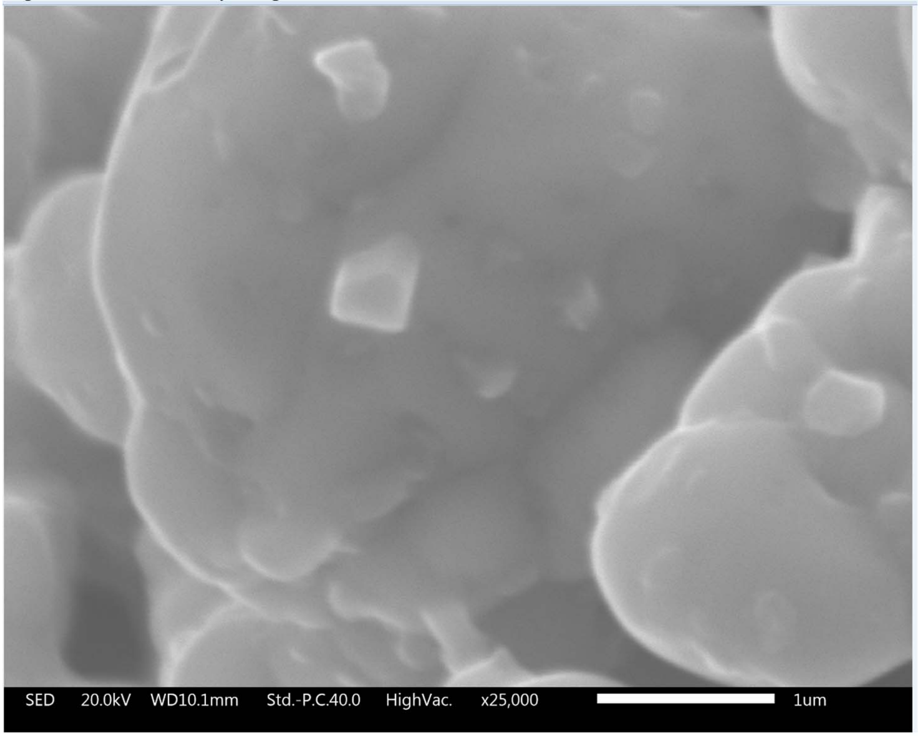

Figure S 3. SEM micrography for Ag_2O.

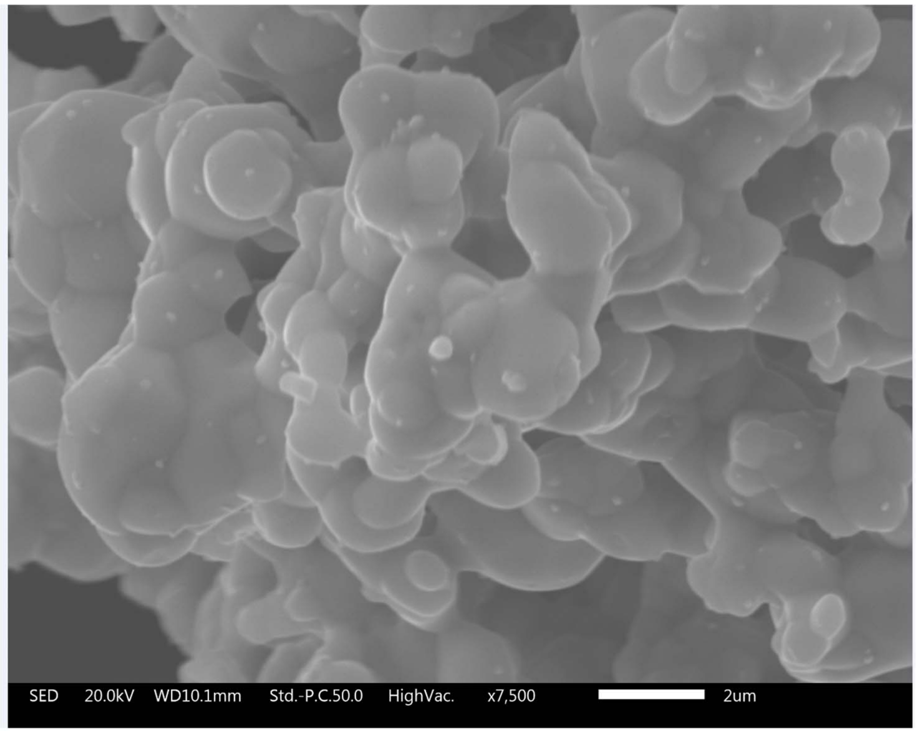

Figure S 4. SEM micrography for Ag_2O.

Supplementary Note 2: Comment on the Biso compared to the literature

The B_O values are similar to what is reported in the literature [2,3], however, the B_{Ag} are 12% to 35% lower when compared to neutron diffraction data and other X-ray sources [2,4,5]. Three main assumptions can explain this discrepancy: (i) the quality of the synchrotron data employed here compared to laboratory sources; (ii) the use of significantly longer Q ranges reported by Wada et al. from Neutron sources; and (iii) the expanded supercell adopted in this work to estimate correlation from more distant atomic pairs, which modelled the subtle TDS features of the data, increasing the quality of the fit. However, the literature itself shows a broad range of values for the DW coefficients and they might have a relation with the source material investigated.

Supplementary Note 3: Laboratory Data Modelling of Ag_2O

The XRPD profile modelling for the data collected with Mo-K_α radiation at RT using the STOE lab equipment is shown in Figure S 5(a). The TDS oscillatory features are visible beneath the main Bragg peaks of the Ag_2O cuprite structure, magnified in Figure S 5(b). Ag peaks are present in the pattern but are attributable to less than 1% of the sample weight fraction (identified by the grey traces in Figure S 5(a)). The peaks corresponding to the silver carbonate phase are modelled with pseudo-Voigt functions, with positions indicated by the blue balls in Figure S 5(a). The modelling of the TDS part is also shown in Figure S 5(b). These TDS features can be clearly separated from the capillary background, scaled with a second-order polynomial function of 2θ and represented with green lines in the plot. The R_{wp} is 4.38%, and the GoF 1.84. Crystallite size and microstrain line profile contributions were modelled by the WPPM approach [1] using an effective microstructural model. The microstrain Warren plots are given in Figure S 6 for the whole range of temperatures and plane directions.

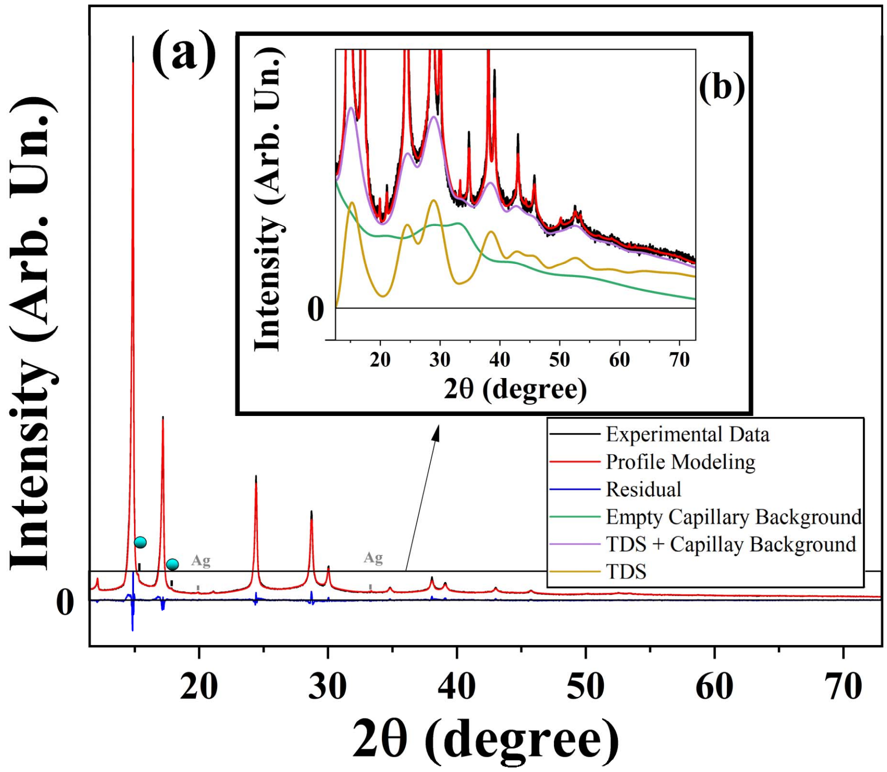

Figure S 5. (a) Profile modelling (red line) of the observed diffraction pattern for the Ag_2O purchased powder (black dots). Residuals are given in blue. Ag peak positions are represented by the grey traces and the silver carbonate peaks by the blue balls. (b) Zoom of the black square region of the item a. The empty capillary contribution is displayed with a green line, the TDS in the dark yellow line, and the TDS + capillary in purple.

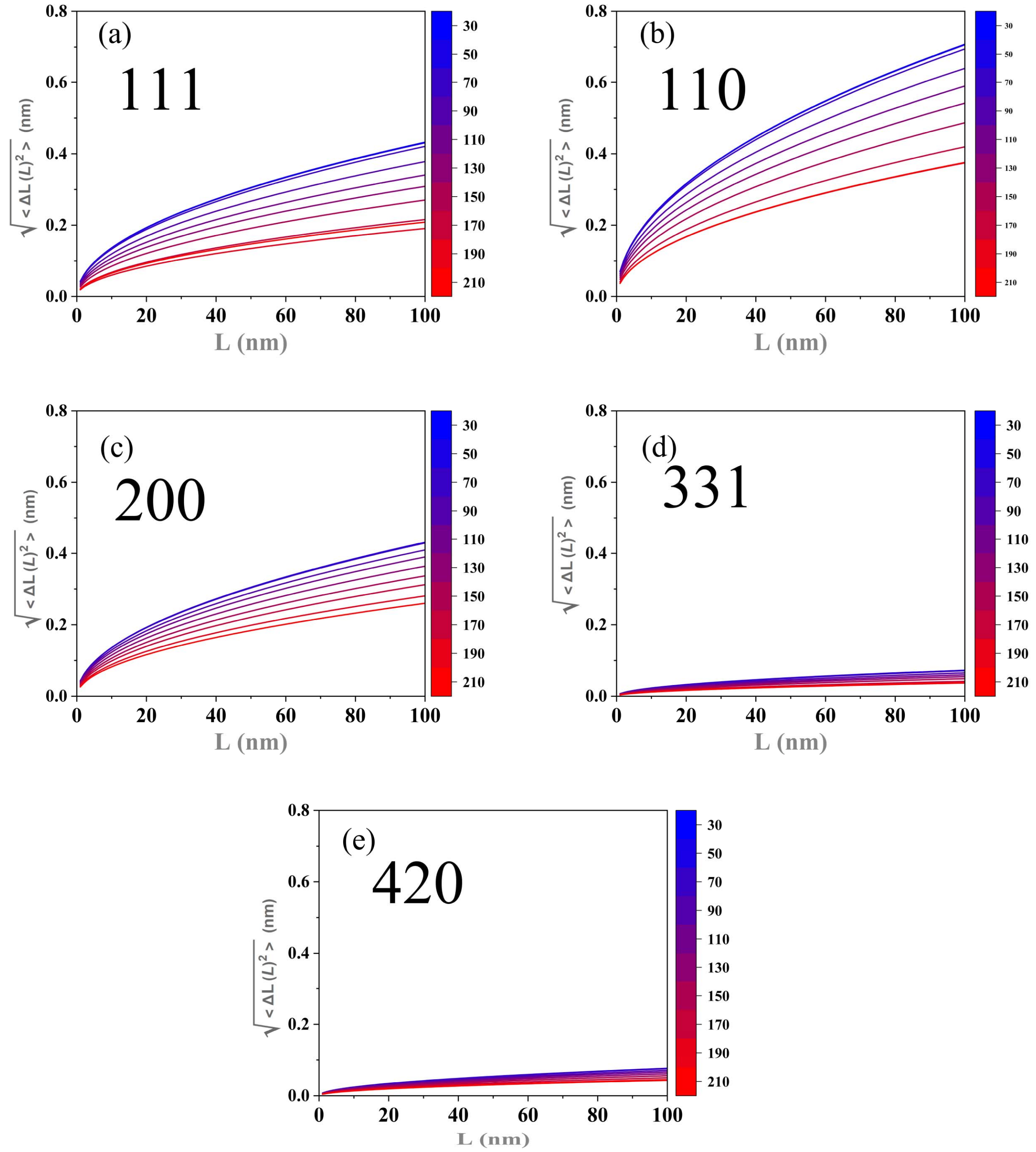

Figure S 6. Waren plots using the PAH model for the purchased powder for the [111],[110],[200],[331], and [420] directions.

Supplementary Note 4: TDS Supercell Modelling

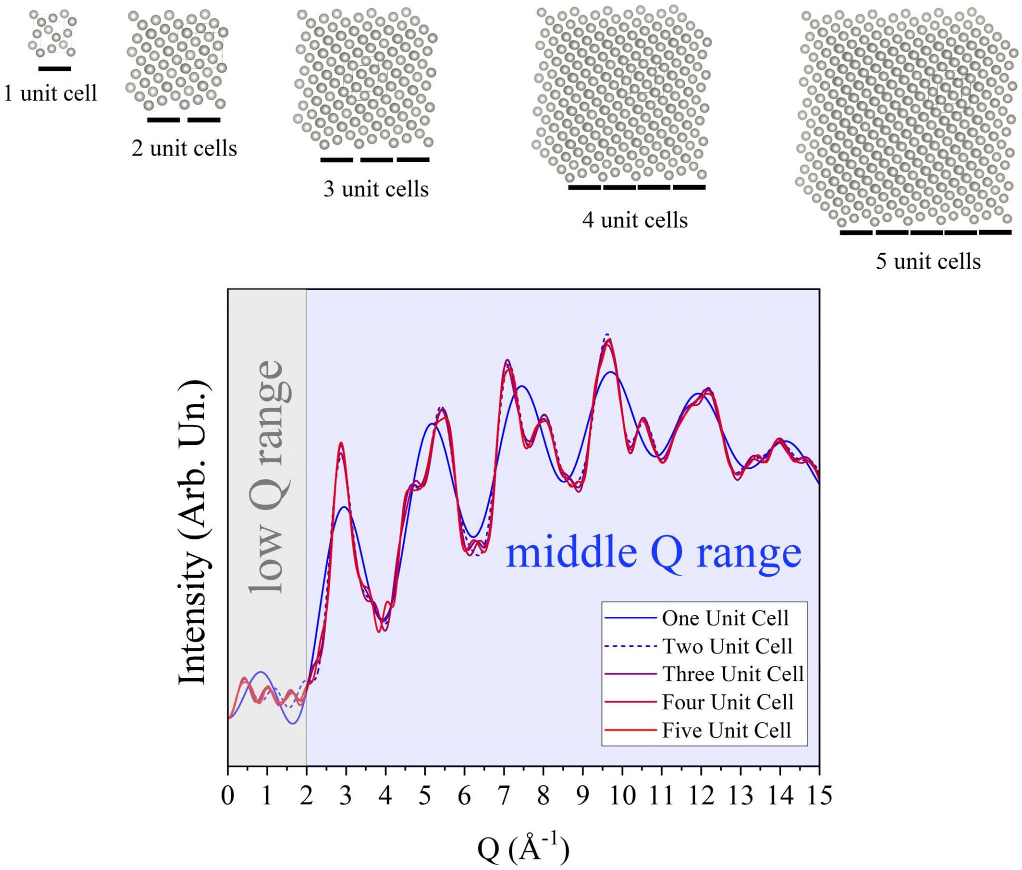

Figure S 7. (Top) representation of the different supercell models for monoatomic fcc Pd. (Bottom) TDS obtained by the Sakuma modelling of simulated Pd via MD (details in Ref. [6]*) using different supercell models.*

From eq.(4), one can understand each of the pair correlation contributions for the TDS as an element of an expanded series in terms of a Sinc$(Q\, r_{ss'})$ function, that adds together to form the total TDS pattern, represented in yellow in FIG. 2(b) and (c) of the main text. With this assumption, limiting the maximum pair distance (r_{max}) in the Sakuma TDS approach introduces ripples at low 2θ ranges (or at low Q), in a similar manner to the Q_{max} cut-off for PDF analysis [7]. This effect is schematically represented in Figure S 7. This makes it imperative to assume that the TDS intensity at the low Q range is zero since it bears no information and can correlate with the background in this region, inducing unphysical negative values for intensity. In the Figure S 7, we highlight how the increase of r_{max} also induces more subtle features in the TDS. However, adding pairs more distant than that of a 3x3x3 supercell does not significantly alter the form of the TDS in mid Q range, making the three-unit-cell model an excellent compromise between reducing the number of parameters and the quality of the fitting.

Supplementary Note 5: Correlation coefficients for the cooling curves

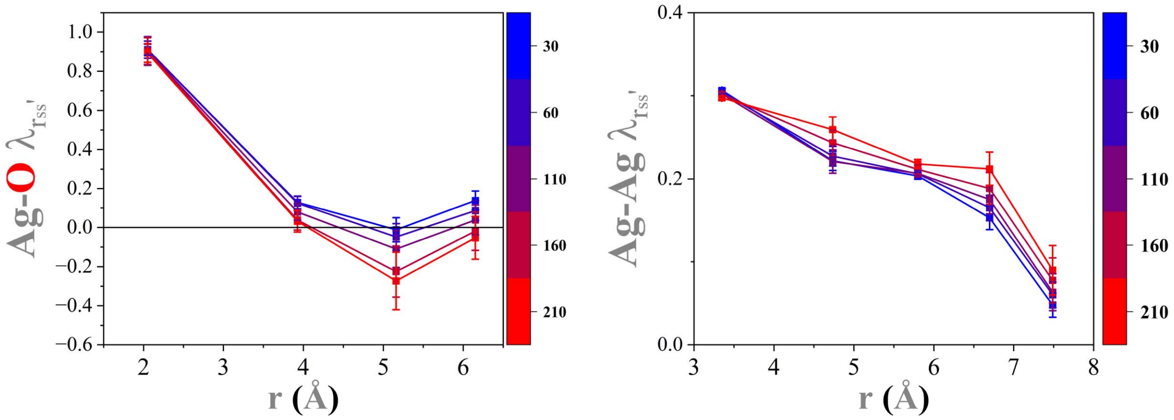

Figure S 8. (left) Ag-O correlation coefficients versus the pair distance r from 210°C to RT (cooling curves). (right) Ag-Ag correlation coefficients versus the pair distance r from 210°C to RT.

Supplementary Note 6: PDF fitting for the AIMD simulated data

Definitions

Here we compute the time-averaged PDF ($g(r)$) from the atomic coordinates retrieved from the AIMD. The procedure is similar to what had been reported in Ref. [6]. The analysis is done for the nearest neighbours of Ag-Ag and Ag-O. In order to fit the assymetry of the peaks, an effective function was chosen:

$$g(r) = \frac{A}{\sigma_1\sqrt{2\pi}} \text{ Exp}\left[-\left(\frac{(r-R)^2}{2\,\sigma_1^2}\right)\left(1-\frac{(r-R)}{R}\frac{\sigma_2^2}{\sigma_1^2}\right)\right] \tag{S 1}$$

Here, A, R, σ_1, and σ_2 are adjustable parameters. This is a slightly modified form of eq.(13) in Ref. [8], adapted to better fit the anisotropy of Ag_2O PDF peaks. Other definitions for the $g(r)$ are available in [9]. The cumulants C_i^* are obtained by:

$$C_i^* = \int_{Peak} r^i\, g(r)\, dr \tag{S 2}$$

Integrated over the peak area. The value of i corresponds to the ith moment of the distribution. The resulting cumulants have no analytical expression and must be calculated numerically for every temperature. In this case, the C_1^* value corresponds to the average mean value of the pair distance, C_2^* the MSRD, C_3^* is the mean cubic relative displacement, a measure of anisotropy of the PDF peak. The relation between the cumulants and the force constants of the potential defined in eq. (6) are given in Ref. [10], and placed here for convenience:

$$\delta C_1^* = -\left(\frac{3b}{a^2}\right) k_B T \tag{S 3}$$

$$C_2^* = \left(\frac{k_B T}{a}\right) + \left(\frac{k_B T}{a}\right)^2 \left[\left(\frac{6b}{a}\right)^2 - \left(\frac{12c}{a}\right)\right] \tag{S 4}$$

$$C_3^* = -\left(\frac{k_B T}{a}\right)^2 \left(\frac{6b}{a}\right) \tag{S 5}$$

$$C_4^* = \left(\frac{k_B T}{a}\right)^3 \left[\frac{108b^2}{a^2} - \frac{24c}{a}\right] \tag{S 6}$$

Here $\delta C_i^* = C_i^*(T) - C_i^*(T = 0\,K)$. These expressions are obtained by integrating the distribution of interatomic distances $\rho(r)$ given the effective one-dimensional potential $V_e(r)$ given in the main text:

$$\rho(r,T) = \frac{\exp[-\beta\, V_e(r)]}{\int \exp[-\beta\, V_e(r)]\, dr} \tag{S 7}$$

Where $\beta = 1/k_B T$. This expression is valid within the classical approximation for sufficiently high temperatures.

Ag-Ag pairs

In addition, cuprite structures are known for containing two distinct types of Ag-Ag second neighbors: (i) Type A, where Ag-Ag pairs share an O atom and are part of the same network, and (ii) Type B, where Ag-Ag pairs belong to different networks. Both types are illustrated in Figures S 9(a) and (c), respectively. For the Type A, the $g(r)$ function is shown in Figure S 9(a) with corresponding cumulants in Figure S 9(b). The peaks exhibit no asymmetry, so C_3^* was not calculated. Figure S 9(c) and (d) display the $g(r)$ function and cumulant fittings for Type B pairs. Fitting parameters for both types are summarized in Table S 1.

Notably, Type B pairs exhibit a slightly shorter pair distance, approximately 0.02 Å less than Type A pairs, which is reflected in a fourfold reduction in local thermal expansion for Type A. This structural distinction also leads to a 50% reduction in the force constant for Type A pairs compared to Type B pairs. Additionally, the median $g(r)$ for Type B pairs decreases with temperature, indicating negative thermal expansion (NTE) behavior. Meanwhile, the first cumulant, C_1^*, shows a linear increase with temperature, driven by significant $g(r)$ broadening as both σ_1 and σ_2 increase with temperature. What we observe in the main text is the sum of both Type A and Type B pairs, that leads to an effective negative local thermal expansion of Ag-Ag pairs.

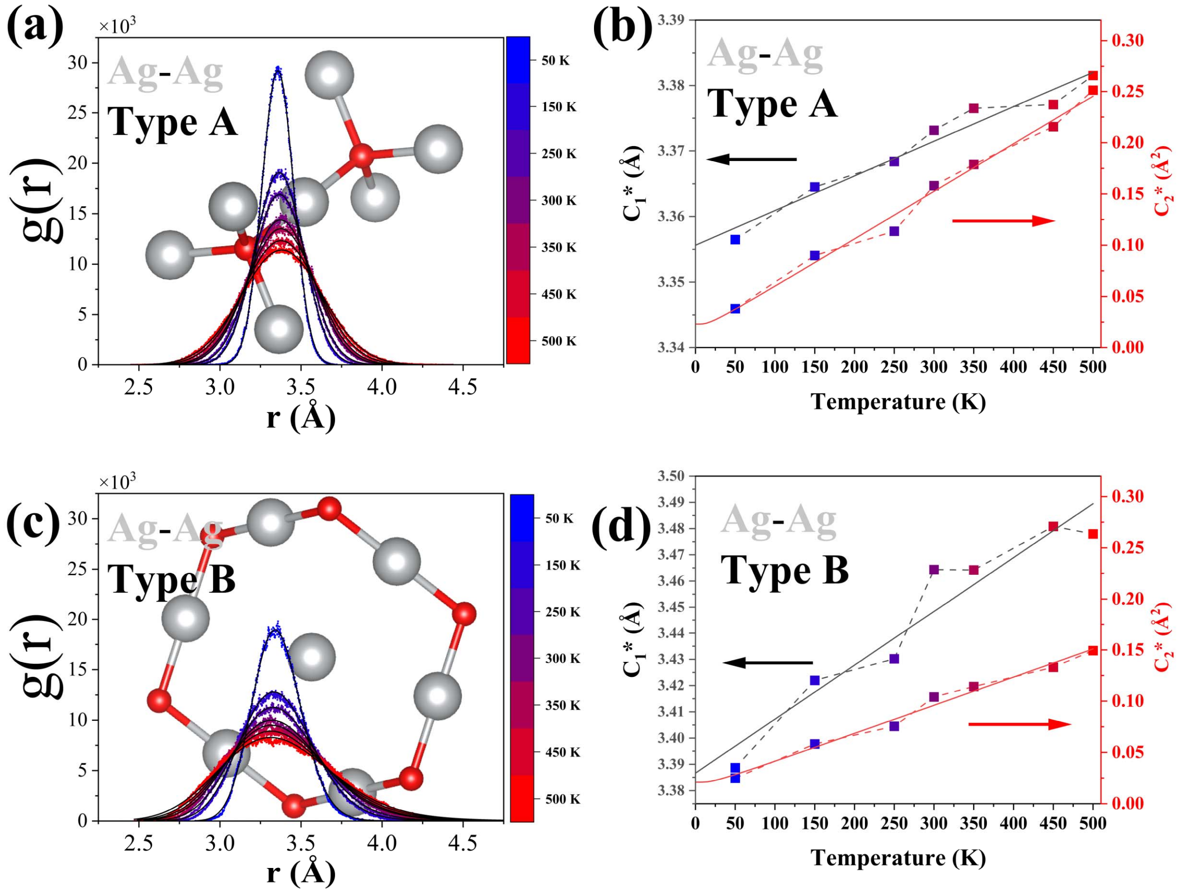

Figure S 9.(a) $g(r)$ of the Type A Ag-Ag pairs. The dots represent simulated values and black the fitting using eq.(S 1) with parameters given in Table S 1. The colors represent the temperatures according to the scale displayed on the left. (b) first (C_1^*) and second (C_2^*) cumulants of the $g(r)$ given by the squared symbols. Black and red dashed lines a mere guide to the eyes and correspond to C_1^* and C_2^* data, respectively. The solid lines represent the fitting using a linear equation for C_1^* and eq.(7) of the main text for C_2^*.

Table S 1.Fitted parameters for the Type A and Type B second neighbors Ag-Ag pairs.

Pair Type	Force Constant a (eV Å^{-2})	Force Constant b (eV Å^{-3})	ω_E (THz)	MSRD at $T = 0\ K$ (Å^2)	θ_E (K)	Local Thermal Expansion (10^{-5} K^{-1})
**Type A**	0.19	0	5.78	0.013	44	1.4
**Type B**	0.31	0.03	7.48	0.013	57	6.1

Comparison for different simulation boxes

In addition, different AIMD simulations were performed using (2×2×2, 3×3×3, and 4×4×4 unit cells) at 300 K to verify if all the dynamics of the system can be described by the 3×3×3 model. In Figure S 10, the $g(r)$ and its fitting for Ag-O and Ag-Ag pair is given. The parameters of fitting are available in Table S 2. The fitting quality is similar for each simulation box, and it but definitely better for models bigger than 3 unit cells, resulting in more precise statistics. Nevertheless, the parameters given in Table S 2 are identical for all the simulations. Since 4×4×4 simulations take a considerable amount of time for its completion, we believe that a 3×3×3 model for Ag2O can be more efficient while describing the necessary dynamics of the system.

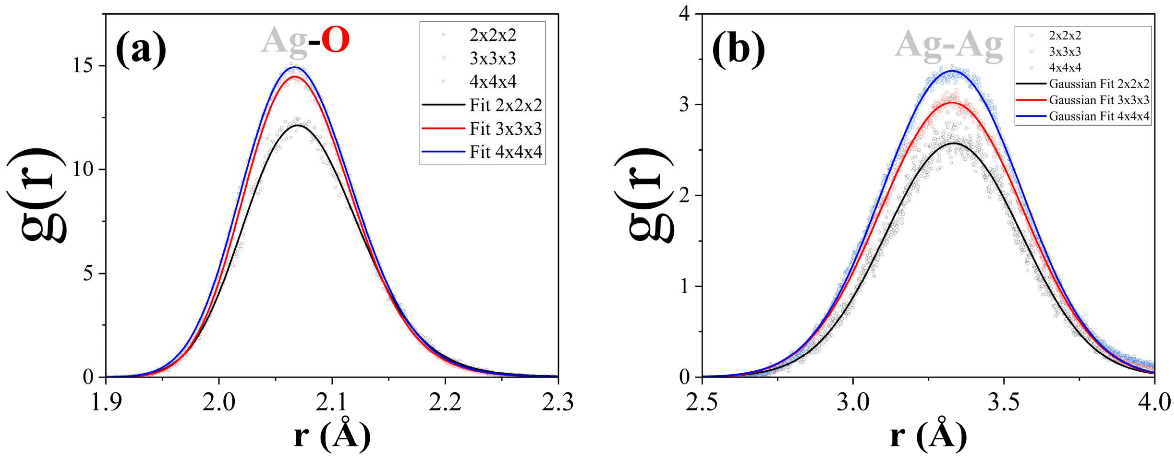

Figure S 10. $g(r)$ simulated data and fitting for the Ag-O first shell (a) and Ag-Ag second-shell (b) for different simulation boxes (2×2×2, 3×3×3, and 4×4×4 unit cells).

Table S 2. Fitting parameters obtained for the graphics of Figure S 10 using eq.(S 1).

Simulation	R-Ag-Ag (Å)	σ_1-Ag-Ag	R-Ag-O	σ_1-Ag-O	σ_2-Ag-O
**2x2x2**	3.3332(3)	0.4538(7)	2.06954(7)	0.05008(5)	0.1012(4)
**3x3x3**	3.3275(2)	0.4752(7)	2.06706(2)	0.04729(2)	0.0968(2)
**4x4x4**	3.281(1)	0.4647(3)	2.06657(2)	0.04878(2)	0.0957(2)

Supplementary Note 7: Equations used for the force constant estimations

The relation between the second-order cumulant of $\rho(r,T)$, MSRD, and the harmonic term, a, is given in the following form for the Wada et al. formulation [2,8,10,11]:

$$MSRD = \frac{k_B T}{a} \quad \text{(S 8)}$$

Where k_B is the Boltzmann constant. Estimations of the force constants via TDS require corrections to those made by Ishikawa *et al.* [12] since the force constants are required to go to zero for $\lambda_{r_{ss'}} \to 0$. It is given in the form:

$$a = 8\frac{\pi^2 k_B T \lambda_{r_{ss'}}}{3(B_s + B_{s'})\left(1 - \lambda_{r_{ss'}}\right)} \quad \text{(S 9)}$$

Table S 3 shows the values of the force constants using the approaches from Wada *et al.* [2] [eq.(S 8)] and Ishikawa *et al.* [13] [eq.(S 9)] including all types of measurements and estimations performed in this work. These values differ significantly from those reported using the Einstein fit and literature values from EXAFS [14]. Both eqs.(S 8) and (S 9) are related to the cumulant expansion of the $\rho(r,T)$, however, they do not correspond to a physical model as eq. (7) of the main text does (Einstein model). In addition, it is important to note that the Einstein model works only for the nearest neighbours, since the force constant ($\mu\omega_E^2$) does not go to zero if the $MSRD(T) - MSRD(T = 0K) \to 0$. For longer pair distances, the Debye model, assuming the collective phonon vibration of the lattice are more appropriate [15]. For this reason, the Ishikawa *et al.* corrections are *ad-hoc* and are not appropriate for distant pairs longer than the second or third coordination shell with exceptional cases [16]. This justifies the big discrepancy in force constant values to the Einstein fit model reported in Table 1 of the main text.

If the latter equations are applied to the room temperature (300 K) trajectories, the Wada model values coincide with the values reported by EXAFS for Ag-O pairs [14], 5.9 eV Å^{-2} (see Table S 3). However, Ag-Ag force constants differ by a factor of two. Therefore, the Ag-O force coefficients match with Ref. [14] must be viewed as a coincidence since EXAFS equations also report values obtained from the Einstein model fit. Again, Ishikawa *et al.* approach of eq.(S 9), the values differ in order of magnitude from experimental values for the Ag-Ag pairs.

Table S 3. Force constants obtained using the correlation coefficient values refined from TDS using laboratory data (Mo), synchrotron data (Sync), PDF refinement (PDF), and AIMD simulations (discussed next). Values are estimated at room temperature.

Model	Ag-O	Ag-Ag
**Force Constant using Wada model (eV Å^{-2})**	MoK$_\alpha$: 1.95<br>Synchrotron: 0.51<br>PDF: 0.86<br>Ref.[2]: 1.37 (T=295 K)<br>AIMD: 5.85	MoK$_\alpha$: 0.31<br>Synchrotron: 0.34<br>PDF: 0.58<br>Ref. [2]: 0.66 (T=295 K)<br>AIMD: 0.48

**Force Constant using Ishikawa (eV Å^{-2})**	MoK$_\alpha$: 0.59<br>Synchrotron: 0.1<br>PDF: 0.24<br>Ref. [2]*: 0.39 (T=295 K)<br>AIMD: 1.9	MoK$_\alpha$: 0.031<br>Synchrotron: 0.024<br>PDF: 0.11<br>Ref. [2]*: 0.14 (T=295 K)<br>AIMD:0.06

**Estimated correction using the values of DW and $\lambda_{r_{ss'}}$ available in the reported works.*

Supplementary Note 8: Interatomic force constants and the mode Gruneisen parameter from DFT (0K).

Interatomic force constants (IFCs):

For phonons within a potential energy given by

$$V[\vec{r}(j_1 l_1), \ldots, \vec{r}(j_n l_N)]$$

where $\vec{r}(j_i l_i)$ is the position vector of the j-th atom in the l-th unit cell, and n and N are the number of atoms in a unit cell and number of unit cells respectively, the force on an atom is given by,

$$F_\alpha(jl) = -\frac{\partial V}{\partial r_\alpha(jl)}$$

where α is the Cartesian index.

The second order interatomic force constant between atoms at positions α and β is then written as,

$$\Phi_{\alpha\beta}(jl, j'l') = \frac{\partial^2 V}{\partial r_\alpha(jl)\partial r_\beta(j'l')} = -\frac{\partial F_\beta(j'l')}{\partial r_\alpha(jl)}$$

where β is the Cartesian index, and j' and l' are the indices of atoms of in a unit cell, and the unit cell itself, respectively.

In the finite displacement method used with DFT, the equation for the force constants is approximated as,

$$\Phi_{\alpha\beta}(jl, j'l') \simeq -\frac{F_\beta\big(j'l'; \Delta r_\alpha(jl)\big) - F_\beta(j'l')}{\Delta r_\alpha(jl)}$$

where $F_\beta\big(j'l'; \Delta r_\alpha(jl)\big)$ are the forces on atoms with a finite displacement $\Delta r_\alpha(jl)$.

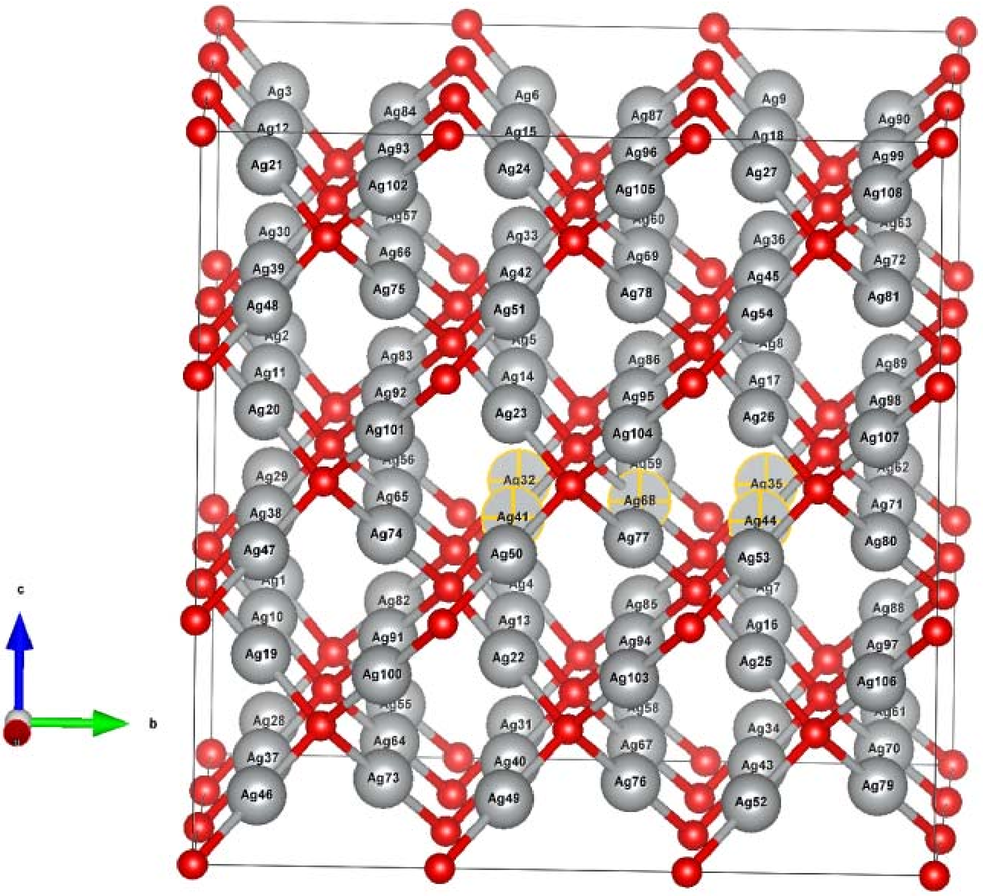

Figure S 11. Nearest neighbor Ag-Ag pairs in the xy-plane, separated by 3.329 Å.

$$\Phi_{\mathrm{Ag68,Ag32}} = \Phi_{\mathrm{Ag68,Ag44}} = \begin{pmatrix} 0.0532 & 0.0248 & -0.005 \\ 0.0248 & 0.0532 & 0.005 \\ 0.005 & -0.005 & 0.0052 \end{pmatrix} eV/\text{Å}^2$$

$$\Phi_{\mathrm{Ag68,Ag41}} = \Phi_{\mathrm{Ag68,Ag35}} = \begin{pmatrix} 0.3255 & 0.3044 & 0.1543 \\ 0.0248 & -0.3255 & -0.1543 \\ -0.1543 & 0.1543 & 0.2546 \end{pmatrix} eV/\text{Å}^2$$

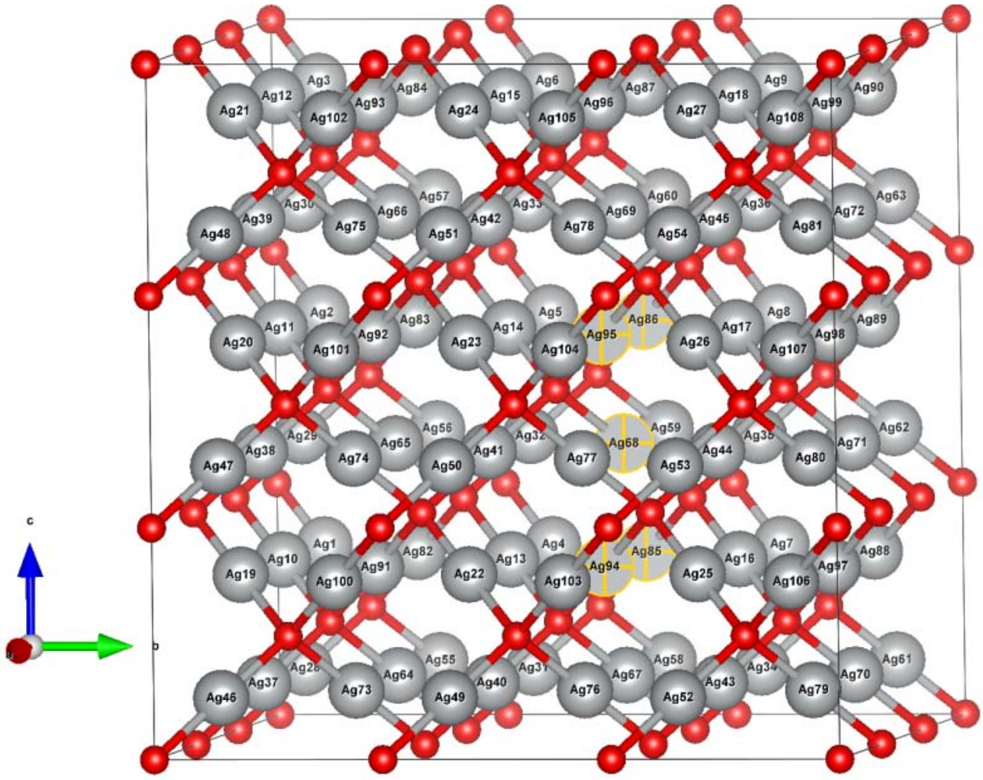

Figure S 12. Nearest neighbor Ag-Ag pairs in the yz-plane, separated by 3.329 Å.

$$\Phi_{\mathrm{Ag68,Ag95}} = \Phi_{\mathrm{Ag68,Ag85}} = \begin{pmatrix} -0.3255 & -0.1543 & -0.3044 \\ 0.1542 & 0.2546 & 0.1543 \\ -0.3044 & -0.1543 & -0.3255 \end{pmatrix} eV/\text{Å}^2$$

$$\Phi_{\mathrm{Ag68,Ag56}} = \Phi_{\mathrm{Ag68,Ag94}} = \begin{pmatrix} 0.0532 & 0.005 & -0.0248 \\ -0.005 & 0.0052 & -0.005 \\ -0.0248 & -0.005 & 0.0532 \end{pmatrix} eV/\text{Å}^2$$

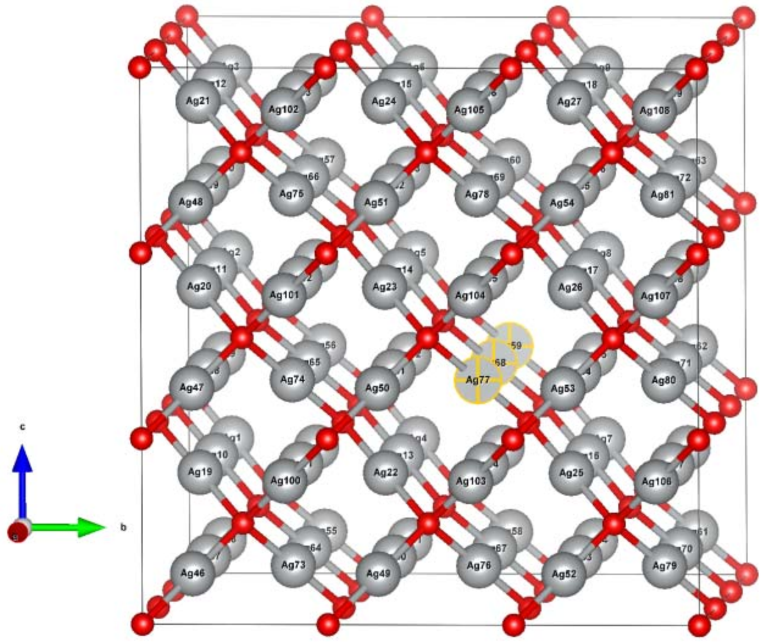

Figure S 13. Second nearest neighbor Ag-Ag pairs, separated by 4.708 Å.

$$\Phi_{\mathrm{Ag68,Ag56}} = \Phi_{\mathrm{Ag68,Ag94}} = \begin{pmatrix} -0.0358 & -0.0127 & 0.0127 \\ -0.0127 & -0.0007 & 0.0135 \\ 0.0127 & 0.0135 & -0.0007 \end{pmatrix} eV/\text{Å}^2$$

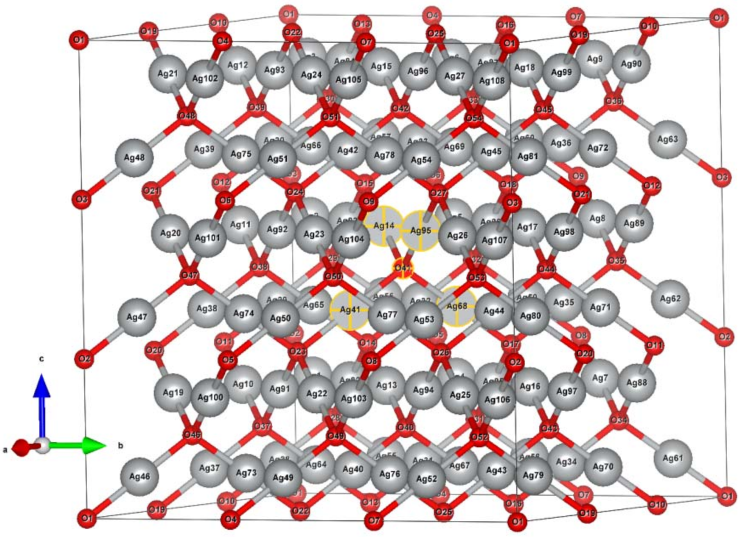

Figure S 14. Nearest neighbor Ag-O pairs, separated by 2.039 Å.

$$\Phi_{\mathrm{Ag95,O41}} = \Phi_{\mathrm{Ag95,O41}} = \Phi_{\mathrm{Ag95,O41}} = \Phi_{\mathrm{Ag95,O41}} = \begin{pmatrix} -3.8952 & -3.3623 & -3.3623 \\ -3.3623 & -3.8952 & -3.3623 \\ -3.3623 & -3.3623 & -3.8952 \end{pmatrix} eV/\text{Å}^2$$

Mode Grueneisen parameter:

The mode Grueneisen parameter $\gamma(\vec{q}_\nu)$ at the wave vector $\vec{q}$ and band index ν is given by,

$$\gamma(\vec{q}_\nu) = -\frac{V}{\omega(\vec{q}_\nu)}\frac{\partial\omega(\vec{q}_\nu)}{\partial V}$$

$$= -\frac{V}{2[\omega(\vec{q}_\nu)]^2}\left\langle \vec{e}(\vec{q}_\nu)\middle|\frac{\partial D(\vec{q})}{\partial V}\middle|\vec{e}(\vec{q}_\nu)\right\rangle$$

where V is the volume, $\omega(\vec{q}_\nu)$ is the phonon frequency, $D(\vec{q})$ is the dynamical matrix, a $\vec{e}(\vec{q}_\nu)$ is the eigenvector. Using the finite difference method, this is approximated as,

$$\gamma(\vec{q}_\nu) = -\frac{V}{2[\omega(\vec{q}_\nu)]^2}\left\langle \vec{e}(\vec{q}_\nu)\middle|\frac{\Delta D(\vec{q})}{\Delta V}\middle|\vec{e}(\vec{q}_\nu)\right\rangle$$

The computed Grueneisen parameter for Ag_2O is given in Figure S 14.

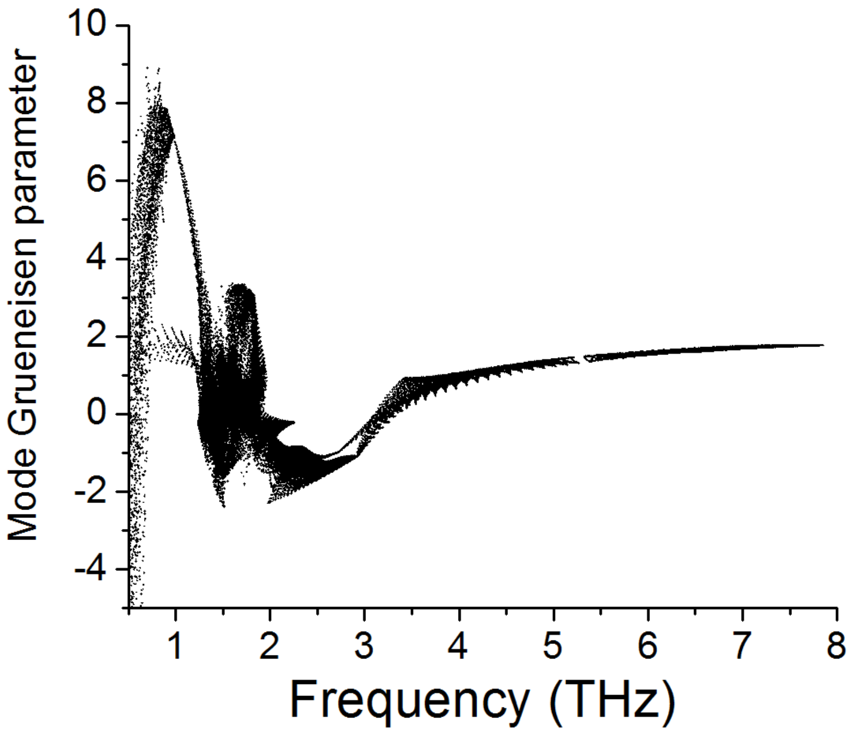

Figure S 15. Calculated mode Grueneisen parameter for Ag_2O.

References

[1] P. Scardi, C.L. Azanza Ricardo, C. Perez-Demydenko, A.A. Coelho, Whole powder pattern modelling macros for TOPAS, J. Appl. Crystallogr. 51 (2018) 1752–1765.

https://doi.org/10.1107/S160057671801289X.
[2] T. Wada, T. Sakuma, R. Sakai, H. Uehara, Xianglian, H. Takahashi, O. Kamishima, N. Igawa, S.A. Danilkin, Inter-atomic force constants of Ag 2O from diffuse neutron scattering measurement, Solid State Ionics. 225 (2012) 18–21. https://doi.org/10.1016/j.ssi.2012.02.007.
[3] T. WADA, R. SAKAI, M. ITAKURA, Y. HAYASHI, M. WATANABE, MAKHSUN, T. SAKUMA, H. TAKAHASHI, S.A. DANILKIN, PRESSURE DEPENDENCE OF CRYSTAL STRUCTURE OF AG2O BY X-RAY DIFFRACTION MEASUREMENT, in: Solid State Ionics, WORLD SCIENTIFIC, 2012: pp. 563–568. https://doi.org/10.1142/9789814415040_0067.
[4] B.J. Kennedy, Y. Kubota, K. Kato, Negative thermal expansion and phase transition behaviour in Ag 2O, Solid State Commun. 136 (2005) 177–180. https://doi.org/10.1016/j.ssc.2005.05.043.
[5] Y. Ishikawa, S.A. Danilkin, M. Avdeev, T. Kamiyama, T. Sakuma, Thermal Expansion Properties of Ag 2 O Crystal Structure by Powder Neutron Diffraction, in: Proc. 2nd Int. Symp. Sci. J-PARC — Unlocking Myster. Life, Matter Universe —, Journal of the Physical Society of Japan, 2015: pp. 1–7. https://doi.org/10.7566/JPSCP.8.031016.
[6] P. Scardi, M.A. Malagutti, Thermal Diffuse Scattering from Nanocrystalline Systems, Cryst. Growth Des. (2024). https://doi.org/10.1021/acs.cgd.3c01507.
[7] E. Takeshi, S.J.L. Billinge, Local Structure of Well-Ordered Crystals and Systems with Competing Interactions, 2012. https://doi.org/10.1016/B978-0-08-097133-9.00008-3.
[8] P. Fornasini, F. Monti, A. Sanson, On the cumulant analysis of EXAFS in crystalline solids, J. Synchrotron Radiat. 8 (2001) 1214–1220. https://doi.org/10.1107/S0909049501014923.
[9] D.S. Yang, S.K. Joo, N. Hilbrandt, Relationship between pair distribution and cumulant expansion for anharmonic vibrational systems, J. Korean Phys. Soc. 33 (1998) 59–65.
[10] P. Fornasini, Study of lattice dynamics via extended x-ray absorption fine structure, J. Phys. Condens. Matter. 13 (2001) 7859–7872. https://doi.org/10.1088/0953-8984/13/34/324.
[11] A. Yoshiasa, K. Koto, H. Maeda, T. Ishii, The Mean-Square Relative Displacement and Displacement Correlation Functions in Tetrahedrally and Octahedrally Coordinated A N B 8-N Crystals, Jpn. J. Appl. Phys. 36 (1997) 781. https://doi.org/10.1143/JJAP.36.781.
[12] Y. Ishikawa, T. Sakuma, T. Hashimoto, Xianglian, O. Kamishima, S.A. Danilkin, Estimation of phonon dispersion relations using correlation effects among thermal displacements of atoms, Atom Indones. 41 (2015) 1–5. https://doi.org/10.17146/aij.2015.349.
[13] Makhsun, T. Hashimoto, T. Sakuma, H. Takahashi, O. Kamishima, N. Igawa, S.A. Danilkin, Estimation of force constants of Al from diffuse neutron scattering measurement, J. Phys. Soc. Japan. 83 (2014) 6–9. https://doi.org/10.7566/JPSJ.83.074602.
[14] G. Artioli, M. Dapiaggi, P. Fornasini, A. Sanson, F. Rocca, M. Merli, Negative thermal expansion in cuprite-type compounds: A combined synchrotron XRPD, EXAFS, and computational study of Cu2O and Ag2O, J. Phys. Chem. Solids. 67 (2006) 1918–1922. https://doi.org/10.1016/j.jpcs.2006.05.043.
[15] G. Dalba, P. Fornasini, R. Grisenti, D. Pasqualini, D. Diop, F. Monti, Anharmonicity effects on the extended x-ray-absorption fine structure: The case of cadmium selenide, Phys. Rev. B. 58 (1998) 4793–4802. https://doi.org/10.1103/PhysRevB.58.4793.
[16] Y. Ishikawa, T. Sakuma, T. Hashimoto, X. Xianglian, O. Kamishima, S.A. Danilkin, Estimation of Phonon Dispersion Relations Using Correlation Effects Among Thermal Displacements of Atoms, Atom Indones. 41 (2015) 1. https://doi.org/10.17146/aij.2015.349.
